# Mouse models to study von Willebrand factor in inflammation: a scoping review

**DOI:** 10.1186/s40635-026-00935-z

**Published:** 2026-06-24

**Authors:** Hassan Masood, Veronica DeYoung, Peter Andrisani, Arthane Kodeeswaran, Taylor Sparring, Jaskirat Arora, Natasha Savic, Jonathan L. Babulic, Davide Matino, Patricia C. Liaw, Colin A. Kretz, Alison Fox-Robichaud

**Affiliations:** 1 Thrombosis and Atherosclerosis Research Institute (TaARI), 237 Barton Street East, Hamilton, L8L 2X2 Canada; 2 Department of Medicine, Queen’s University, Kingston, Canada; 3 Department of Medicine, Faculty of Health Sciences, McMaster University, Hamilton, Canada; 4 Division of Critical Care, Department of Medicine, Faculty of Health Sciences, McMaster University, Hamilton, Canada; 5 Hamilton Health Sciences, Hamilton, Canada

**Keywords:** Inflammation, von Willebrand factor, Mouse Models, ADAMTS13

## Abstract

**Background:**

VWF is released from activated endothelial cells and activated platelets in response to vascular injury, and is now recognized as an important contributor to a growing number of inflammatory conditions. This scoping review aims to identify and evaluate mouse models that have been used to study von Willebrand Factor (VWF) in the context of inflammation. Understanding the role of VWF in these models is crucial for selecting appropriate models and developing effective targeted treatments.

**Methods:**

A comprehensive literature search was conducted to identify studies using mouse models of inflammation from inception to October 2024. Two reviewers independently screened and extracted data on inflammation model methodology, organ outcomes, histological analyses, and biochemical changes if they pertain directly to VWF, or indirectly through ADAMTS13.

**Results:**

150 studies published between 2000 and 2024 met inclusion criteria; 25% utilized C57BL/6 mice, and 43% used only male mice. Most (63%) studies used acute inflammation models, and 56 (37%) studies induced inflammation chemically in mice. Most studies (75%) reported an increase in VWF antigen levels, while only 31% of studies reported an increase in VWF activity. Few studies (33%) have also highlighted the therapeutic potential of ADAMTS13 to significantly reduce inflammation.

**Conclusions:**

There is variability in the methods and outcomes in mouse models of inflammation. Future studies should consider the impact of methodology on VWF-related outcomes when using these models to study VWF-related processes and therapeutics.

**Supplementary Information:**

The online version contains supplementary material available at https://doi.org/10.1186/s40635-026-00935-z.

## Introduction

Inflammation is a fundamental biological response to tissue injury or infection, essential for host defense and tissue repair [1]. Despite medical advancements, inflammation-driven morbidity and mortality remain high, [2] underscoring the importance of understanding vascular and molecular mediators that regulate inflammatory injury.

Von Willebrand factor (VWF), a large multimeric glycoprotein best known for its role in hemostasis, is increasingly recognized as an important mediator of inflammation and thromboinflammation [3–6]. Inflammatory cytokines such as TNF-α and IL-1β stimulate endothelial Weibel–Palade body exocytosis, releasing VWF and P-selectin to the endothelial surface, where they promote leukocyte rolling and platelet adhesion [7–9]. In vitro studies using cultured endothelial cells have shown that cytokine stimulation increases VWF secretion and expression of adhesion molecules [10]. In vivo models of endotoxemia, ischemia-reperfusion, and autoimmune inflammation demonstrate that elevated VWF and reduced ADAMTS13 activity exacerbate microvascular thrombosis and organ injury [11–13]. Collectively, these findings position VWF as a key mediator linking thrombosis and inflammation

Under physiological conditions, VWF multimers are cleaved by ADAMTS13 (A Disintegrin and Metalloprotease with Thrombospondin motifs 13) to regulate their size and activity [3,5]. During inflammation, reduced ADAMTS13 activity leads to the accumulation of ultra-large VWF multimers with enhanced platelet-binding capacity [3]. This imbalance in the VWF–ADAMTS13 axis has been documented across several inflammatory conditions, including ischemia-reperfusion injury, autoimmune disease, and sepsis [3].

Experimental models remain instrumental in defining these mechanisms. Murine models enable controlled manipulation of VWF, allowing study of its roles in inflammation and thrombosis [14]. However, interspecies differences in endothelial biology, immune regulation, and VWF structure can limit translational validity of findings [14–17].

The recent approval of recombinant ADAMTS13 for thrombotic thrombocytopenic purpura (TTP) marks an important shift in therapeutic options for targeting VWF in different disease settings [18]. Therefore, the present scoping review aims to bridge current literature gaps by identifying all mouse models used to study VWF in inflammation, outlining their suitability for this purpose.

## Methods

### Protocol and registration

This scoping review adheres to the PRISMA Extension for Scoping Reviews (PRISMA-ScR) using the JBI framework for scoping reviews [19]. The study was a retrospective analysis of previously published work, and therefore, approval from the ethics board was not required. The protocol for this study was published in Open Science Framework and can be accessed with the identifier: https://doi.org/10.17605/OSF.IO/N8VC7.

### Research question

What are the mouse models that have demonstrated mechanistic involvement of VWF and/or ADAMTS13 in the context of inflammation?

### Search strategy

A literature search was conducted using MEDLINE, Web of Science, and EMBASE to identify primary studies of mouse models of inflammation, thrombosis, and hemostasis that were used to study VWF from inception to October 2024. The primary search terms were von Willebrand factor, VWF, ADAMTS13, ADAMTS-13, mice, and mouse. Associated subheadings and available term expansions were applied in each database (see Supplemental Digital Content 1).

### Eligibility criteria and selection process

All citations from the initial search were uploaded into Covidence (Veritas Health Innovation, Melbourne, Australia)[20]. A trial of 25 randomly selected studies were initially screened by each investigator to pilot the screening process for both title-abstract and full-text stages.

*Inclusion criteria*: Studies were included if they used a mouse model of inflammation, or a disease state with secondary inflammation measures; there were no restrictions to the sex, strain, or age of mice; there were no restrictions to mouse models narrower than previously stated inclusion criteria (e.g., models of both arterial and venous thrombosis were included); and the study had a direct outcome measure of VWF or ADAMTS13 involvement or activity. Only primary studies with an in vivo model were. For this review, “mechanistic involvement” was defined as studies providing direct functional evidence of VWF or ADAMTS13 participation in the inflammatory process. This included use of VWF-deficient or ADAMTS13-deficient mice, pharmacological inhibition or exogenous administration of VWF or recombinant ADAMTS13, or demonstration of a causal relationship between VWF/ADAMTS13 and an inflammatory outcome.

*Exclusion criteria*: Excluded were studies involving species other than mice; not a model of inflammation, or a disease state associated with VWF or ADAMTS13; no direct outcome measure of VWF or ADAMTS13; or VWF or ADAMTS13 measured only by gene expression. Publications such as editorials, abstracts, commentaries, letters, systematic reviews, and meta-analyses were excluded.

### Data extraction

Additional methodological details, including data extraction templates, and screening calibration procedures, are provided in the Supplementary Digital Content 3, in line with PRISMAScR reporting guidance.

## Results

### Study selection

**Fig. 1:**
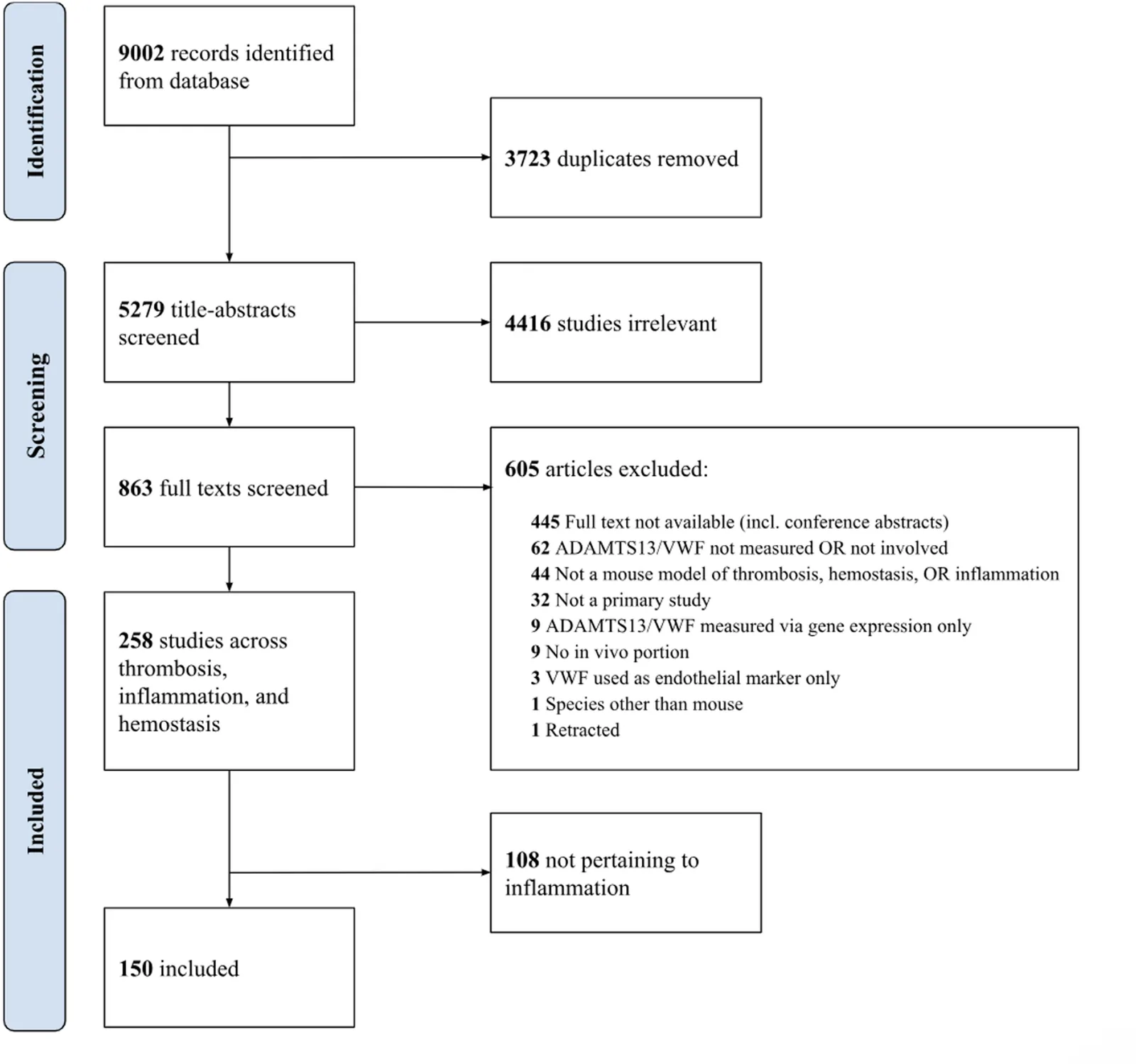
Preferred Reporting Items for Systematic Reviews and Meta-Analyses (PRISMA) Flow Chart

As shown in Fig. 1, the search strategy yielded 5279 studies after de-duplication. Title-abstract and full-text screening yielded 258 studies pertaining to thrombosis, hemostasis, or inflammation for data extraction. Among these studies, 150 employed inflammation models and were therefore included in this review. All included studies and their detailed characteristics are outlined in Supplemental Table S1.

### Study characteristics

**Fig. 2:**
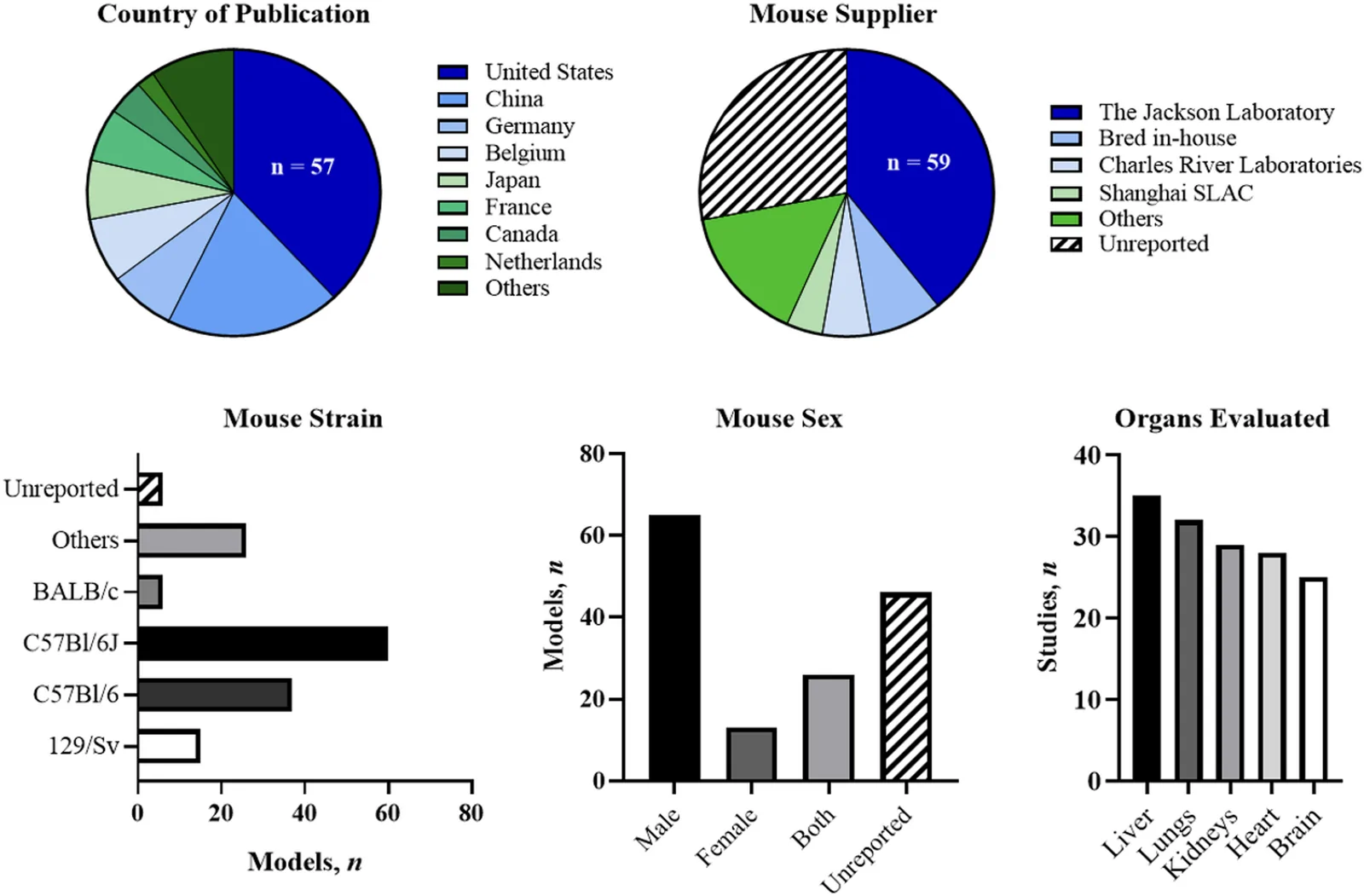
Summary of study characteristics. Pie charts represent proportions across all included studies, whereas mouse strain and mouse sex bar graphs pertain to all included models, of which there may be more than one per study. Unreported proportions are depicted with hatch marks

Figure 2 summarizes the characteristics of the 150 included studies. Studies were published between 2000 and 2024 from 20 different countries, predominantly the United States of America (38%). Most studies (40%) used a C57BL/6J background with 4% not reporting it. Most studies (43%) used only male mice, 31% did not report the sex of mice, 13 studies (9%) used only female mice, and 26 studies (17%) used both male and female mice. Most mice came from commercial suppliers. 59 studies (39%) obtained their mice from the Jackson Laboratory; however, 12 studies (8%) used in-house bred mice, and 42 studies (28%) did not report the source. The liver was the most investigated organ (23%), followed by the lungs (21%), the kidneys (19%), the heart (19%), and the brain (17%). Most of the studies (88%) reported the age of mice, with significant variation across studies.

### Models of inflammation

**Fig. 3:**
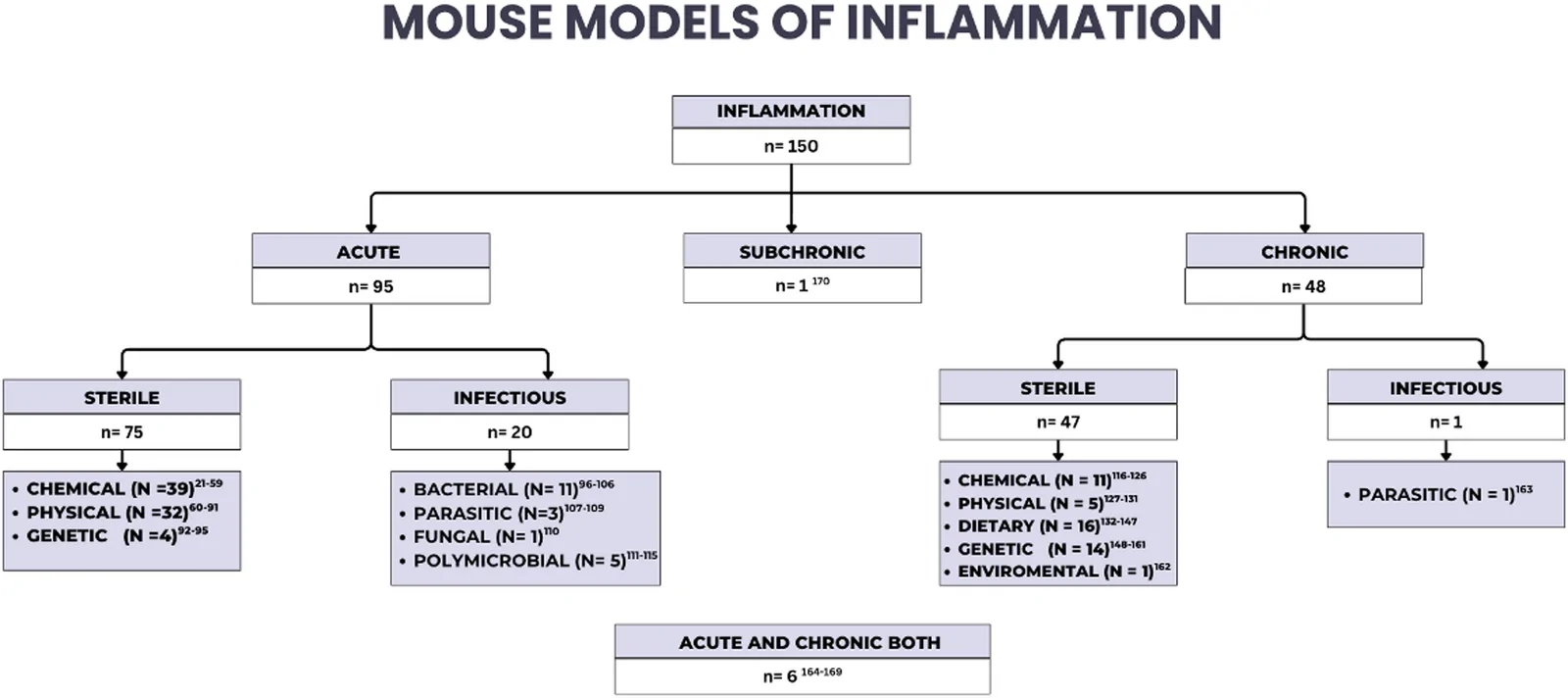
Summary of inflammation model characteristics. Flow diagram showing the distribution of mouse inflammation models categorized as acute, sub-chronic, or chronic, and further subdivided by sterile and infectious etiologies

Figure 3 summarizes the characteristics of inflammation models used to study VWF across the included studies. Details about triggers used to induce inflammation are provided in Supplemental Tables S2. Among included studies, 63% employed acute inflammation models [21–115], 32% used chronic models [116–163], 4% incorporated both [164–169], and one study focused on sub-chronic inflammation.^170^]. Classification of inflammation models was based on study duration: acute models assessed outcomes within ≤ 7 days, sub-chronic models within 8–28 days, and chronic models > 28 days after challenge [ These models were further subcategorized as sterile or infectious. 75 studies (50%) employed acute sterile inflammation [21–95], whereas 20 studies (13%) used acute infectious inflammation. [96–115].

### VWF and VWF-mediated processes

Table 1 presents a synthesis of the outcome measures reported across the included studies investigating VWF in the context of inflammation (see Supplementary Table S3 for a detailed version). The most frequently reported outcomes were increased VWF antigen levels and VWF-related proteins (e.g. ADAMTS13, VAMP3, SNAP23, and/or Factor VIII), documented in 75% and 74% of studies, respectively. Endothelial dysfunction and inflammatory markers were also commonly assessed (66% and 63% studies respectively). Inflammatory markers primarily included circulating cytokines, such as IL-6, TNF-α, IL-1β, IL-1α, IL-12 (p40), IL-2, IFN-γ, and IL-4, whereas endothelial dysfunction was assessed using markers of endothelial activation, including P-selectin and ICAM-1. The therapeutic potential of ADAMTS13 was explored in 33% of studies, with findings suggesting an overall benefit of administering rhADAMTS13 to reduce vascular inflammation, and plaque formation in atherosclerosis. Other reported outcomes included VWF multimer distribution (33%), platelet aggregation (34%), and neutrophil infiltration (37%).

**Table 1:** Outcomes of inflammation pertaining to VWF and VWF-mediated processes

Outcome Measure	Studies Reporting, *n* (%)	Key Findings
↑ VWF Antigen	112 (75)	Increased VWF antigen indicates a prothrombotic and inflammatory state
↑ VWF Activity	46 (31)	Functional role for VWF in amplifying inflammatory responses
VWF Multimer Distribution	49 (33)	Accumulation of ultra-large VWF multimers
Platelet Adhesion	64 (43)	VWF-mediated platelet adhesion may contribute to inflammation
Endothelial Dysfunction	99 (66)	Insights into the role of VWF in endothelial function
Inflammatory Markers	95 (63)	Increase in proinflammatory cytokines (IL-6, TNF-α, and IL-1β)
VWF-Related Proteins	111 (74)	ADAMTS13 modulates inflammatory plaque progression via VWF. VAMP3 and SNAP23 are critical for VWF secretion. Factor VIII; Acute-phase protein; antigen levels rise during inflammation, contributing to pro-thrombotic state.
Therapeutic potential of ADAMTS13	50 (33)	ADAMTS13 reduces vascular inflammation, suggesting its potential as a therapeutic target across several disease models (e.g. Acute kidney injury, traumatic brain injury, ischemia-reperfusion injury, atherosclerosis, sepsis)
Leukocyte recruitment	66 (44)	VWF promotes leukocyte recruitment
Neutrophil Infiltration	55 (37)	Associated with elevated VWF expression
Platelet aggregation	51 (34)	Increased aggregation correlated with elevated VWF antigen levels and pro-inflammatory conditions

## Discussion

VWF, a large plasma adhesive glycoprotein, exerts a pivotal role in inflammation [171]. However, a shift from the dominant understanding of inflammation and thrombosis as distinct pathways to the novel concept of immunothrombosis has led to inconclusive outcomes [171]. A prior review [172] assessed inflammatory mechanisms across diverse animal models and their limitations. This scoping review systematically identifies and evaluates murine models used to investigate the role of VWF during inflammation, with a focus on methodological consistency, outcome variability, and translational relevance.

### VWF Antigen Elevation Is Common but Function Is Undercharacterized

Across the 150 included studies, the most consistent finding was an elevation of VWF plasma antigen levels: 112 of 135 studies that measured VWF antigen (83%) reported an increase, one study documented a significant decrease following polytrauma compared to sham controls (108.1 vs. 186.7 ng/mL, *p* < 0.001), [61] and two studies used VWF-deficient models in which plasma VWF measurement was not applicable [41,139]. This near-universal antigen elevation across both sterile and infectious triggers suggests that endothelial activation and Weibel–Palade body exocytosis are a common final pathway [3,26,38,42]. In contrast, VWF activity was reported in only 31% of studies, and changes in multimer distribution in 33%, reflecting a systematic under-investigation of VWF function relative to VWF abundance. Where VWF activity and multimer composition were assessed, results were more variable: in some sterile injury models, VWF antigen increased despite depletion of high-molecular-weight multimers and limited proportional platelet-mediated pathology, [26,109] whereas in infectious or autoimmune contexts antigen elevation was accompanied by accumulation of ultra-large multimers, reduced ADAMTS13 activity, and clear functional consequences that were reversible with recombinant ADAMTS13, [117] indicating that antigen elevation alone is an insufficient surrogate for VWF-mediated pathology. The discordance between antigen and activity data represents the most critical unresolved question in this field: it remains unclear whether elevated VWF in many models is functionally consequential or a bystander marker of endothelial perturbation [26,109]. Future studies should measure both antigen and activity in parallel, alongside multimer distribution, to resolve this gap.

### Temporal classification of inflammatory models: strengths and limitations

A temporal framework for classifying models as acute (≤ 7 days), sub-chronic (8–28 days), or chronic (> 28 days) was adopted in this review because it provides a reproducible, operationally transparent classification applicable across heterogeneous triggers. We acknowledge, however, that duration-based classification has limitations: the same inducing agent (e.g., bleomycin) may be studied at acute or chronic timepoints, and the biological features captured will differ accordingly. Importantly, the temporal category reflects when outcomes were assessed, not the intrinsic biology of the trigger. Investigators should therefore consider both the temporal phase and the nature of the inducing agent when selecting a model for a specific research question.

### Justification for inclusion of chronic atherosclerosis models

The inclusion of atherosclerosis models warrants direct comment. Atherosclerosis is a chronic inflammatory arterial disease in which VWF has an established mechanistic role in endothelial activation, platelet recruitment, and plaque progression;^132,135,139^ its inclusion is therefore consistent with the review’s scope of VWF-mediated inflammation. We nonetheless recognize that atherosclerosis may not be considered an inflammatory model from a critical care standpoint, and findings should be interpreted within that context and not extrapolated to acute inflammatory conditions without qualification.

### Methodological heterogeneity limits cross-study comparisons

There were significant discrepancies in methodological approaches, as outlined in Table 2. Variability in study outcomes included VWF multimer distribution and VWF-related proteins, such as factor VIII (FVIII), ADAMTS13, VAMP3 and SNAP23, platelet adhesion and aggregation, and proinflammatory cytokines like IL-6 and TNF-α, as well as measures of endothelial and organ dysfunction. Methodological variations, including inflammation induction methods, endpoints, and outcome measures (biomarkers, histology, survival, etc.), complicated direct comparisons between studies.

**Table 2:** Gaps in knowledge and potential solutions

Research Gap	Description	Implications for Future Research
*Limited assessment of VWF activity and multimer distribution*	VWF activity and multimer composition have been under-studied in inflammation, limiting understanding of their functional impact.	Evaluate both VWF activity and multimer profiles across inflammatory models to clarify mechanistic links
*Lack of longitudinal studies*	Most studies are cross-sectional, lacking long-term follow-up data.	Conduct longitudinal studies to assess the long-term effects of inflammation on VWF antigen levels and activity
*Underexplored therapeutic potential of ADAMTS13*	Limited research on the therapeutic applications of ADAMTS13 in inflammation.	Future studies should explore the therapeutic potential of ADAMTS13 in restoring the ADAMTS13–VWF imbalance in inflammatory and thrombo-inflammatory conditions, with particular focus on improving microvascular thrombosis, endothelial dysfunction, and organ injury outcomes.
*Mechanistic understanding of VWF-mediated platelet aggregation*	VWF-dependent and fibrinogen-dependent pathways driving aggregation in inflammatory conditions remain poorly characterized	Investigate VWF-mediated versus other platelet activation pathways in inflammation and clarify their contributions to thrombo-inflammatory outcomes in vivo.
*Sex as a biologic variable*	Most studies did not use both male and female mice.	Include both male and female mice in studies to understand sex differences in inflammatory responses and VWF activity.
*Age as a biologic variable*	Significant variation in the age of mice used (3 weeks to 80 weeks)	Harmonize age selection criteria to improve comparability and understand age-related differences in inflammatory responses.

]Older mice exhibit a heightened inflammatory response due to age-related changes in immune function, observed across several included models [61,114,170,173,174]. Age-related differences in VWF expression and function have also been documented [175]. VWF antigen levels rise significantly with increasing age, reflecting heightened endothelial activation and possibly diminished clearance [176]. Consequently, studies conducted in young mice may underestimate the contribution of VWF to disease processes that predominantly affect older individuals. This age-related variability is important because VWF release is closely tied to endothelial activation in inflammation [177].

]Studies have shown that male and female mice can exhibit different immune responses, which can affect the severity and progression of inflammation [178,179]. For example, estrogen has been shown to modulate VWF antigen levels and activity, which could influence the results of studies on VWF in inflammation [137,140,180]. Estrogen has also been shown to downregulate the production of pro-inflammatory cytokines and upregulate anti-inflammatory cytokines [181]. Furthermore, studies show higher neutrophil counts and increased neutrophil recruitment to sites of inflammation within male mice along with more active NF-κB signaling compared to female mice [179,182]. This sexual dimorphism calls for the need for both sexes in inflammation research. Future studies investigating VWF in the context of inflammation should include both male and female mice as a minimum standard, consistent with the NIH policy on sex as a biological variable. Where single-sex designs are scientifically justified, the rationale must be explicitly stated. Studies should also report hormonal status where relevant, particularly in female mice, given the established modulatory effects of estrogen on VWF secretion and activity. Reporting sex-stratified outcomes, even descriptively, would substantially improve the interpretability and cross-study comparability of findings in this field.

The variability of inflammation among the studies diminished the translational applicability of the findings. Acute inflammation models, such as those induced by lipopolysaccharide (LPS), provide insights into innate immune responses but may not accurately reflect chronic inflammatory conditions [183]. Chronic models, better mimic long-term inflammatory diseases but are more complex and time-consuming to establish [184]. Chronic inflammation models are particularly relevant for studying diseases like atherosclerosis and rheumatoid arthritis, where VWF plays a significant role [185,186]. Studies also show sustained endothelial activation and continuous secretion of VWF following chronic inflammation [187]. Different methods of inducing inflammation (chemical, dietary, or genetic) can also lead to varying inflammatory responses, relevant for understanding VWF dynamics. Chemical agents like lipopolysaccharide (LPS) induce acute inflammation, characterized by a rapid release of pro-inflammatory cytokines such as IL-1, IL-6, and TNF-α [183]. This acute response can lead to a transient but significant increase in VWF antigen levels. In contrast, high-fat or high-sugar diets mimic chronic low-grade inflammation seen in metabolic disorders like obesity and type 2 diabetes, leading to sustained elevation of inflammatory markers and VWF antigen levels [188,189]. The route of inducing inflammation, intraperitoneal (IP) versus intravenous (IV), can influence the distribution, kinetics, and severity of the inflammatory response, which is critical for accurately modelling inflammatory diseases. IP administration is commonly used due to its ease and consistency; while IP-injected molecules do reach systemic circulation, early local exposure can affect organ-specific cytokine responses and the timing of peak systemic cytokine levels compared with IV administration [190,191] IV administration, while more technically challenging, provides a more direct route to systemic inflammation and may be more relevant for conditions like sepsis [192,193]. The choice of route should reflect the specific inflammatory pathways under investigation.

The endpoints used to assess inflammation varied across studies, including measures of cytokine levels, histopathological changes, and survival. Modern animal research guidelines, including the ARRIVE 2.0 reporting standards, discourage using death as an endpoint; instead, studies should report the use of humane endpoints, such as the Murine Sepsis Score, to determine when animals are euthanized [194,195]. Not all studies included in this review specified how survival was assessed, highlighting variability in adherence to ethical and methodological standards. The choice of endpoints should also consider the specific aspects of inflammation being studied, such as vascular permeability, leukocyte recruitment, and cytokine production. Additionally, certain analgesic agents can influence inflammatory responses and should be reported and standardized to ensure that pain management does not confound the experimental outcomes. Some agents, including pentobarbital sodium and isoflurane, have immunomodulatory effects that may influence inflammatory and immunohistochemical endpoints, limiting translational findings [196].

### Timing of ADAMTS13 intervention and translational implications

Among the 33% of studies that explored the therapeutic potential of ADAMTS13, the available evidence favors post-injury (therapeutic) administration over prophylactic use. Studies administering recombinant ADAMTS13 after the onset of ischemia-reperfusion injury, endotoxemia, or organ injury consistently reported reductions in microvascular thrombosis, inflammatory cytokine levels, and organ damage scores,[67,96,98,117,127] whereas prophylactic administration was less uniformly beneficial and, in some models, did not reach statistical significance for primary outcomes [109,132]. This pattern suggests that the therapeutic window for ADAMTS13 is linked to the accumulation of ultra-large VWF multimers and the propagation of thrombo-inflammation following the initial insult, rather than prevention of the insult itself [54,56,117]. Nevertheless, definitive conclusions regarding timing are constrained by heterogeneous dosing, routes, and outcome measures across studies. Standardized dose-response and timing experiments within established models are needed before clinical translation of ADAMTS13 as an anti-inflammatory therapeutic can be responsibly pursued.

### Reporting standards and model selection: recommendations for the field

Given the breadth of mice strain and sex reporting deficiencies identified in this review, we propose that such a checklist should prioritize three domains. First, complete animal characterization: strain and sub-strain designation, source, sex, age, and housing conditions should be mandatory minimum reporting items, as each independently influences VWF expression, endothelial activation, and inflammatory responses. Second, standardized VWF outcome measurement: studies should report VWF antigen, activity, and multimer distribution in parallel wherever possible, rather than selecting a single measure; use of WHO-referenced standards and validated activity assays should be specified. Third, humane endpoint and intervention reporting: the endpoint criterion used, the analgesic and anesthetic agents employed (with doses), and the timing and route of any VWF- or ADAMTS13-directed intervention must be stated explicitly. Adoption of these standards, ideally integrated into the ARRIVE 2.0 framework, would substantially enhance reproducibility and cross-study comparability in this field.

Dietary variability across studies can also affect inflammation outcomes, complicating interpretation. Many studies vaguely describe diets as “normal” or “standard chow,” which can lead to substantial differences in nutrient composition and caloric intake [197,198]. These variations can affect the gut microbiota, metabolic responses, and overall inflammatory status of the mice [198]. For instance, refined diets often lack soluble fiber, which is crucial for maintaining a healthy gut microbiota and preventing dysbiosis [199]. Therefore, consistency in diet composition is essential for reproducibility and accurate translation to humans.

Majority of the studies used C57BL/6 mice. Although this is the current gold standard in murine inflammation studies, C57BL/6 mice are not the most appropriate model for all types of inflammatory conditions [200]. Studies have shown that BALB/c and C57BL/6J mice exhibit more pronounced inflammatory responses compared to C57BL/6 N mice, likely due to the upregulation of proinflammatory cytokines such as TNF-α and CXCL1 in response to double-stranded RNA (dsRNA) [201]. Whereas, C57BL/6 N mice show a more attenuated response, including selective induction of IL-1β [201]. Based on evidence synthesized in this review, C57BL/6J mice are appropriate for most models of acute sterile inflammation and ischemia-reperfusion injury, given their well-characterized immune phenotype and broad availability of transgenic lines [26,67]. For studies requiring robust Th2-skewed or allergic inflammatory responses, BALB/c mice are preferable, as they exhibit stronger Th2 polarization and more pronounced airway inflammation than C57BL/6J [36,118] C57BL/6 N mice, which display attenuated cytokine responses to pattern-recognition receptor ligands relative to C57BL/6J, are better suited to studies where a less reactive inflammatory baseline is required, or where VWF-specific effects must be distinguished from background inflammatory noise [84,130]. Investigators should explicitly justify strain selection and consistently report sub-strain designations (J vs. N), as these differences can materially affect VWF-related outcomes and confound cross-study comparisons [130].

To contextualize the methodological variability identified in this review, Table 3 summarizes the evidence base for VWF and ADAMTS13 across six major inflammatory disease contexts derived directly from the 150 included studies. For each category, the table reports the number of studies, the most commonly used models, quantitative VWF outcome data, and the principal knowledge gap, making explicit where the evidence is robust and where it is absent. This synthesis is intended to guide model selection and prioritize areas for future investigation, without prescribing a single recommended model where the data do not support such a conclusion. Across these contexts, mechanistic evidence is uneven. It is strongest for ischemia-reperfusion and cardiac injury models, which provide the largest ADAMTS13 therapeutic dataset; recombinant ADAMTS13 was tested in 88% of these studies and was consistently protective after injury. Sepsis and endotoxemia models are the most numerous. In these, VWF antigen elevation, reduced ADAMTS13 activity, and rhADAMTS13-mediated organ protection have been demonstrated across both sterile and infectious triggers. Evidence is intermediate for atherosclerosis and metabolic inflammation, where VWF has an established role in plaque progression and chronic endothelial activation but activity and multimer data are largely absent. It is sparsest for traumatic brain injury and trauma (*n* = 3), the only context in which VWF antigen decreased, and for autoimmune and neuroinflammatory models (*n* = 2). These represent the largest gaps relative to their clinical relevance.

**Table 3:** VWF and ADAMTS13 evidence by inflammatory disease context

Disease context (*n* studies)	Most common models used	VWF antigen: studies measuring / ↑ / ↓	VWF activity / multimers / ADAMTS13 therapeutic (*n*, %)	Key finding from included studies	Principal knowledge gap
Sepsis / Endotoxemia (*n* = 24) [3,38,53,58,96–114,163]	LPS endotoxemia (*n* = 10); CLP polymicrobial sepsis (*n* = 5); bacterial / parasitic / fungal infection (*n* = 9)	16/24 measured; ↑16, ↓0 (100% of those measuring showed increase)	Activity: 8/24 (33%); multimers: 10/24 (42%); ADAMTS13 therapy: 7/24 (29%)	Consistent VWF antigen elevation across sterile (LPS) and infectious (CLP, S. aureus) triggers; where measured, ADAMTS13 activity is reduced and rhADAMTS13 attenuates organ injury	VWF antigen not measured in 8/24 studies; functional (activity, multimer) data absent in majority; strain not reported in 13/24
Ischemia-Reperfusion / Cardiac Injury (*n* = 16) [11,12,60,63,64,67,70–72,78,80,88,90,91,127,158]	Myocardial I/R (*n* = 4); renal I/R (*n* = 3); cerebral I/R / stroke (*n* = 3); other I/R (*n* = 6)	11/16 measured; ↑11, ↓0 (100% of those measuring showed increase)	Activity: 4/16 (25%); multimers: 4/16 (25%); ADAMTS13 therapy: 14/16 (88%)	Largest ADAMTS13 therapeutic evidence base in this review (88%); rhADAMTS13 consistently protective post-injury across cardiac, renal, and cerebral models; VWF antigen elevation universal where measured	VWF activity and multimer data absent in 75% of studies, despite being the most therapeutically studied context; mechanistic basis for ADAMTS13 benefit incompletely defined
Atherosclerosis (*n* = 8) [129,132,135, 138,139,142,143,146]	ApoE−/− or LDLR−/− on high-fat / Western diet (all 8 studies)	6/8 measured; ↑6, ↓0 (100% of those measuring showed increase)	Activity: 2/8 (25%); multimers: 1/8 (12%); ADAMTS13 therapy: 1/8 (12%)	ADAMTS13 deficiency (Adamts13−/− ApoE−/−) accelerates plaque formation; endothelial-derived VWF drives early lesion development; VWF antigen consistently elevated in plaque-prone models	Lowest functional VWF characterization of any category; strain not reported in 7/8 studies; ADAMTS13 as therapeutic agent virtually unexplored in this context
Metabolic Inflammation — obesity / diabetes (*n* = 7) [119,134,136,137,140,162,188]	High-fat diet / diet-induced obesity (*n* = 4); STZ-induced diabetes (*n* = 2); hyperglycemia (*n* = 1)	6/7 measured; ↑6, ↓0 (100% of those measuring showed increase)	Activity: 0/7 (0%); multimers: 3/7 (43%); ADAMTS13 therapy: 1/7 (14%)	VWF antigen universally elevated across dietary and pharmacological models; however no study in this category measured VWF activity, leaving the functional consequence of chronic VWF elevation in metabolic disease entirely uncharacterized	VWF activity completely unmeasured (0/7); strain not reported in any study; ADAMTS13 as therapeutic target virtually unexplored
Traumatic Brain Injury / Trauma (*n* = 3) [61, 64, 71]	TBI — fluid percussion / acrolein (*n* = 2); polytrauma + hemorrhagic shock (*n* = 1)	3/3 measured; ↑2, ↓1 (plasma VWF decreased post-polytrauma vs. sham: 108.1 vs. 186.7 ng/mL, *p* < 0.001)	Activity: 1/3 (33%); multimers: 1/3 (33%); ADAMTS13 therapy: 2/3 (67%)	Only context in this review where VWF antigen decreased; conflicting direction of response between TBI (increase) and polytrauma (decrease) demonstrates that VWF is not uniformly upregulated across all acute inflammatory injuries	Very small evidence base (*n* = 3); conflicting VWF directionality; mechanistic basis for decrease in polytrauma not established
Autoimmune / Neuroinflammation (*n* = 2) [31,117]	DSS colitis (*n* = 1); Toxoplasma gondii encephalitis (*n* = 1)	1/2 measured; ↑1, ↓0	Activity: 0/2 (0%); multimers: 0/2 (0%); ADAMTS13 therapy: 2/2 (100%)	Both studies tested ADAMTS13 therapeutically and reported benefit; no VWF activity or multimer data in either study; evidence base is insufficient for conclusions	Critical underrepresentation (*n* = 2 across all autoimmune models); represents the largest unaddressed gap given clinical relevance; VWF functional characterization entirely absent

### Limitations

This scoping review was limited to English-language publications, which may have resulted in the exclusion of relevant studies. In addition, the review focused exclusively on murine models, limiting generalizability to other species and clinical contexts. Finally, inclusion was restricted to studies with full-text availability, which may have excluded otherwise relevant publications.

## Conclusion

Methodological variability across murine models used to study VWF in inflammation has contributed to heterogenous findings. These discrepancies, including variations in inflammation induction methods, endpoints, and the use of analgesia, have resulted in conflicting data that hinder consensus and applicability. Collaborative efforts to establish uniform practices across laboratories are essential for enhancing comparability. By addressing these methodological challenges, future research can better translate preclinical findings into clinical applications. Ultimately, improving therapeutic strategies for inflammatory diseases.

## Supplementary Information

Supplementary Material 1

## Data Availability

The dataset(s) supporting the conclusions of this article are included within the article and its additional file(s). Detailed data extraction tables supporting the findings of this scoping review are available in Supplementary file. No new primary data were generated or analysed in this study.

## Declarations

### Take-home message

This scoping review identifies substantial methodological variability across mouse models used to study von Willebrand factor (VWF) in inflammation, helping explain inconsistent and sometimes conflicting findings in the literature. By highlighting gaps in model selection, reporting practices, and outcome measurement, this review provides a framework to improve the translational relevance of future studies investigating VWF-mediated inflammatory processes.

### 140-character Tweet

Mouse models used to study VWF in inflammation vary widely, creating inconsistent findings. Our review maps these gaps to guide better, more translational research.
