# Correction: mouse models to study von Willebrand factor in inflammation: a scoping review

**DOI:** 10.1186/s40635-026-00971-9

**Published:** 2026-09-09

**Authors:** Hassan Masood, Veronica DeYoung, Peter Andrisani, Arthane Kodeeswaran, Taylor Sparring, Jaskirat Arora, Natasha Savic, Jonathan L. Babulic, Davide Matino, Patricia C. Liaw, Colin A. Kretz, Alison Fox-Robichaud

**Affiliations:** 1 Thrombosis and Atherosclerosis Research Institute (TaARI), 237 Barton Street East, Hamilton, L8L 2X2 Canada; 2 Department of Medicine, Queen’s University, Kingston, Canada; 3 Department of Medicine, Faculty of Health Sciences, McMaster University, Hamilton, Canada; 4 Division of Critical Care, Department of Medicine, Faculty of Health Sciences, McMaster University, Hamilton, Canada; 5 Hamilton Health Sciences, Hamilton, Canada

**Correction: ICMx 14**,** 80 (2026)**

**https://doi.org/10.1186/s40635-026-00935-z**

Following publication of the original article [1], the name of author Patricia C. Liaw has been corrected.

The original article [1] has been updated.
