# Supplementary material for: Mouse models to study von Willebrand factor in inflammation: a scoping review
Source: Intensive Care Med Exp. 2026 Jun 24;14:80. doi: 10.1186/s40635-026-00935-z (PMC13294430; doi:10.1186/s40635-026-00935-z)
Supplement: Supplementary file 1 — Supplementary Material 1 [file 40635_2026_935_MOESM1_ESM.docx]

**Supplemental Digital Content 1.** Search terms adapted for MEDLINE:

1. von Willebrand Factor/

2. VWF.mp.

3. von Willebrand Factor*.mp.

4. ADAMTS13 Protein/

5. ADAMTS13.mp.

6. ADAMTS-13.mp.

7. mice.mp.

8. mouse.mp.

9. exp Mice/

10. or/1-6

11. or/7-9

12. and/10,11

13. limit 12 to English

14. limit 13 to (comment or editorial or letter or meta analysis or “review” or “systematic review”)

15. 13 not 14

**Supplemental Digital Content 2.** Selection process

Conflicts were discussed, and the inclusion and exclusion criteria were refined accordingly. Two independent reviewers then performed title-abstract screening for relevance, followed by full-text review. Conflicts were resolved through discussion or input from a third reviewer. Reasons for exclusion at the full-text stage were recorded, and deduplication was performed automatically through Covidence or manually if it was missed. Each study was tagged as “inflammation”, “thrombosis”, and/or “hemostasis” depending on the model used and outcomes measured. Studies may have more than one tag.

**Supplemental Digital Content 3.** Key data extraction variables

Key information from the included studies was extracted, including

1. Author(s)
2. Title,
3. Year of publication,
4. Country of publication
5. Study objectives (e.g., therapeutic, mechanistic, diagnostic, etc.)
6. Animal model strain (e.g., sex, weight, age, source)
7. Model methodology, including use of humane endpoints
8. Interventions and comparators, including names and dosing, if applicable
9. Outcome measures involving VWF or ADAMTS13 (e.g., clinical signs, measurements taken, protein activity, etc.) and associated relevant methodology

Data extraction was conducted independently and in duplicate by two reviewers using an Excel (Microsoft, Washington, USA) template developed *a priori.* The extraction form was first pilot tested using 20 studies extracted by two independent reviewers, and modifications were made within the template accordingly. Following extraction, discrepancies were resolved through discussion or third-party arbitration. Inter-rater reliability was ensured by having a 20% overlap of included studies independently reviewed and compared.

For this review, extracted data was filtered in Excel using the “inflammation” tag to identify all studies that used a model of inflammation. The remaining studies identified by these methods (i.e., thrombosis and hemostasis models) will be published in separate reviews.

**Supplementary Table S1. Study characteristics**

| **Author** | **Country** | **Mouse Strain** | **Supplier** | **Sex** | **Age of mice (weeks)** | **Tissues Evaluated** |
| --- | --- | --- | --- | --- | --- | --- |
| Plautz 2021^61^ | USA | C57BL/6 | NR | M | “Adult” | Kidneys |
| Wohner 2021^21^ | Netherlands | C57BL/6J | Jackson | M and F | 8–12 | Liver |
| Xu 2021^62^ | China | C57BL/6J | NR | M | 6-8 | aorta, lung, brain, heart, liver, kidney, and spleen. |
| Meng 2021^22^ | China | C57BL/6 | Cavens | NR | 8–12 | Kidneys |
| Xiao 2021^23^ | USA | C57BL/6 | In-house | NR | 8–12 | Lungs, liver, and brain. |
| Nguyen 2021^24^ | France | C57BL/6 | Jackson | F | 8 | Blood (for platelet counts and plasma levels) |
| Poole 2021^164^ | USA | C57Bl/6J | Jackson | F | 9-13 | Liver |
| Shentu 2021^63^ | USA | C57BL/6 | NR | NR | 20-25 | Heart |
| Na 2020^97^ | Sweden | NMRI & C57BL/6 | Envigo & Charles River | F | 6-8 | Kidneys |
| Cui 2021^64^ | China | C57BL/6J | Fourth Military Medical University | M | 8-12 | Heart, liver, kidney, intestine, and lungs |
| Zhou 2021^127^ | China | C57BL/6 | Shanghai SLAC | M | 8-12 | Kidneys |
| Zhu 2020^66^ | China | C57BL/6 | NR | NR | 8–12 | Brain |
| Ozawa 2020^132^ | USA | C57BL/6 | NR | NR | 20 - 30 | Lungs, liver, and Kidneys |
| Xiao 2020^25^ | USA | C57BL/6 | NR | NR | 8–12 | Endothelial Cells |
| Groeneveld 2020^26^ | USA | C57BL/6J | Jackson | M | 8-14 | Liver |
| Wong 2020^65^ | USA | BALB/c and C57BL/6J | Jackson and In-house | M | 8-12 | Skin |
| Peetermans 2020^98^ | Belgium | C57BL/6 | In-house | NR | 5-11 | Liver and Lungs |
| Denorme 2021^67^ | Belgium | C57BL/6 | NR | NR | 10 | Brain |
| Jin 2020^116^ | China | NR | NR | M | 6 | Brain |
| Kraisin 2020^109^ | Belgium | C57BL/6 | Jackson | M and F | 6-7 | Liver and Lungs |
| Tahir 2020^54^ | Germany | C57BL/6 | Charles River, Jackson | M | NR | Cremaster muscle |
| Michels 2020^133^ | Canada | C57BL/6J | Jackson | M and F | 5 | Inferior vena cava and thrombi |
| Lu 2020^117^ | China | C57BL/6 | Shanghai | F | 8 | Spinal Cord |
| Yang 2020^134^ | China | C57BL/6J | Shanghai SLAC and Jackson | M | 8 | Liver |
| Kang 2019^161^ | USA | C57BL/6 | NR | M | 2 to 17 months | Lungs |
| Liesenborghs 2019^103^ | Belgium | C57BL/6 | NR | M and F | 10–15 and >72 | Heart |
| Latifi 2019^55^ | USA | C57BL/6 | NR | NR | 15-20 | Heart and kidneys |
| Ono 2019^68^ | Japan | C57BL/6 | Japan SLC and Jackson | M | 8-12 | Kidneys |
| Horioka 2019^69^ | Japan | C57Bl/6 | NR | M | 8 | Spleen, liver, kidneys, heart, and lungs |
| Chen 2019^27^ | China | C57BL/6 | Hebei Medical University | NR | 8 | Lungs |
| Sorvillo 2019^28^ | USA | C57BL/6J | Jackson | M and F | 3-4 | Mesenteric venules |
| Kraisin 2019^108^ | Belgium | C57BL/6J | Jackson | M and F | 6-8 | Lungs |
| Suraj 2019^118^ | Poland | BALB/c | Medical University of Bialystok | F | 7-8 | Spleen, liver, kidneys, heart, lungs, and brain. |
| Zhou 2019^70^ | China | C57Bl/6 | NR | M | 8-12 | Kidneys |
| Wu 2018^71^ | China | C57Bl/6J | Jackson | M | 12-16 | Brain and Lungs |
| Moccetti 2018^72^ | USA | C57Bl/6 | NR | NR | 20-25 | heart |
| Nicolay 2018^73^ | Germany | C57Bl/6J and C57Bl/6 | Jackson and Charles River | M | 4 to 5 months | Kidneys |
| Urisono 2018^74^ | Japan | C57Bl/6 | Japan SLC and Jackson | M | 8-12 | Liver |
| Witsch 2018^128^ | USA | C57Bl/6J | Jackson | NR | 3-17 | Heart |
| Doddapattar 2018^135^ | USA | C57Bl/6J | NR | F | 6-25 | Heart |
| Sun 2017^29^ | China | C57Bl/6 | Jackson | NR | 6-8 | Liver |
| Kiouptsi 2017^150^ | Germany | C57BL/6J WT and B6;129S4-F8  tm1Kaz  /J | Jackson | M | 8-14 | Liver and Lung |
| Ayme 2017^30^ | France | C57BL/6J | NR | NR | 3-7 | Skin |
| Joshi 2017^165^ | USA | C57Bl/6J | Jackson | M and F | 8-14 | Liver |
| Dhanesha 2017^119^ | Japan | C57Bl/6J | NR | M | 6-8 | Kidneys |
| Claes 2017^104^ | Belgium | C57Bl/6 | NR | NR | 6-8 | Intestine |
| Geys 2017^136^ | Belgium | C57Bl/6J x 129X1/Sv x CASA/RK | NR | M | 5+ | Liver |
| Kim 2017^75^ | South Korea | C57Bl/6J | Orient and Jackson | M | 6-8 | Kidneys |
| Zitomersky 2017^31^ | USA | C57Bl/6J | In-house | M | 6-8 | Colon |
| Chen 2017^151^ | USA | 129/SV × C57BL/6 | Haig Kazazian and University of Pennsylvania | NR | NR | Spleen |
| Alflen 2017^110^ | Germany | 129/Sv/Pas | NR | NR | NR | Lungs |
| O'Regan 2016^107^ | Ireland | C57Bl/6J | Jackson | NR | 8-10 | Brain |
| Zhang 2016^32^ | China | C57Bl/6 | Peking University | M | 6-8 | Lungs |
| Zhu 2016^76^ | China | C57Bl/6J | Shanghai SLAC | M | 10-12 | Brain |
| Michels 2016^168^ | Canada | C57Bl/6J | NR | NR | 9–55 | Endothelial Cells and Blood |
| Adam 2016^120^ | France | C57Bl/6 | genOway | NR | 4-12 | Blood, skin, and mesenteric vessels |
| Ostertag 2016^169^ | USA | C57Bl/6 | Jackson | NR | 2 - 3 months | Plasma and vascular system |
| Liesenborghs 2016^99^ | Belgium | C57Bl/6 | NR | NR | NR | Heart |
| Dincel 2016^163^ | Turkey | Swiss Albino Mice | Saki Yenilli Experi-mental Animal Production Laboratory | F | 12-16 | Brain |
| Bauer 2015^159^ | Germany | Ret transgenic and C57BL/6 | In-house and Jackson | F | 10 | Skin and tumour microvessels |
| Xiang 2015^125^ | USA | C57Bl/6J | Jackson | NR | 8 | lungs, heart, kidneys, and brain |
| Cai 2015^77^ | China | NR | Shanghai SLAC | M | NR | Brain |
| Hung 2015^137^ | Taiwan | Balb/cA | National Laboratory Animal Center | M | 3 | Heart |
| Shim 2015^152^ | USA | C57Bl/6 | NR | NR | 10, 20, 30, 40 | Thoracic Aorta |
| Bayat 2015^92^ | Germany | C57Bl/6J | NR | M | 6-10 | Lungs |
| Rhieu 2014^153^ | USA | C57Bl/6J | Harvard Medical School | NR | “Adult” | Lungs and bone marrow |
| Claes 2014^100^ | Belgium | C57Bl/6 | NR | NR | 6-8 | mesenteric circulation. |
| Savchenko 2014^78^ | USA | C57Bl/6J | Jackson | M | 8-10 | Heart |
| Hilgruber 2014^33^ | Germany | C57Bl/6J | Harlan | NR | 6-10 | Skin |
| Pappelbaum 2013^101^ | Germany | C57Bl/6 | Jackson | NR | 15-20 | postcapillary and collecting venules |
| Mojiri 2013^162^ | Canada | Transgenic | NR | NR | NR | Lungs, heart, liver, brain, and kidney |
| JayakumarAmirtharaj 2014^34^ | India | C57Bl/6 | NR | M and F | NR | Liver |
| Gandhi 2012 - 1^79^ | USA | C57Bl/6J | Jackson | M | 8-10 | Heart |
| De Meyer 2012^80^ | USA | C57Bl/6J | Jackson | M | 8-10 | Heart |
| Jin 2012^143^ | USA | C57BL/6J | Jackson | NR | 6-12 | Aorta |
| Fujioka 2012^90^ | Japan | SV129 | In-house | M | 8-10 | Brain and blood |
| Gandhi 2012 - 2^144^ | USA | C57Bl/6J | in-house | M and F | 6 -16 | Aorta and aortic sinus |
| Delignat 2012^154^ | France | C57BL/6 | Cécile Denis | NR | 8 | Spleen |
| McCarty 2010^155^ | USA | C57BL/6 | NR | NR | 20-30 | Aorta |
| Huang 2010^102^ | USA | C57Bl/6J -CAST/Ei | Jackson | NR | NR | Kidney |
| Patel 2010^111^ | USA | C57Bl/6 | Jackson | M | NR | Muscle |
| Petri 2010^35^ | Germany | C57Bl/6 | NR | NR | 6-8 | Muscle |
| Fujioka 2010^91^ | Japan | SV129 | In-house | M | 8-10 | Brain and Blood |
| Dieude 2009^36^ | Canada | BALB/c | Harlan Sprague Dawley | F | 10-12 | Brain |
| Chauhan 2008^95^ | USA | C57BL/6J/129×1/SV and C57BL/6J | Jackson | M and F | 9-11 | mesenteric veins and skin tissue |
| Noubade 2008^37^ | USA | C57Bl/6J | Jackson | NR | NR | Brain and spinal cord |
| Chauhan 2008^106^ | USA | 129 × 1/Sv, C57BL/6 | Jackson | M and F | 6-30 | Blood and vascular systems |
| Chung 2008^105^ | USA | DBA/2 | Jackson | NR | 9 | Plasma, Liver, and Endothelial Cells |
| Lerolle 2009^112^ | France | C57Bl/6 | Janvier | M | 22 | Kidneys and liver |
| Patel 2008^58^ | USA | C57Bl/6J | Jackson | M | NR | Cremaster muscle venules |
| Mimuro 2008^38^ | Japan | C57Bl/6 | SLC | M | NR | Liver, lung, and kidney. |
| Iwaki 2006^138^ | Ireland | C57Bl/6J | Jackson | M | 12 - 72 | Hearts, Aortic arteries and aortic sinus |
| Bonnefoy 2006^56^ | France | *Tsp1*-null Swiss | NR | NR | 4-5 | Mesenteric venules, Cecum venules and arterioles, and Intestinal blood vessels |
| Delignat 2007^156^ | France | 129/- | In-house | M and F | 7-10 | Blood |
| Kallas 2007^157^ | Estonia | 129 S6/Sv | In-house | M | 8-10 | Blood |
| EspiritoSanto 2004^160^ | Netherlands | MX1Cre+LRPflox/floxLDLR–/–APOE–/– | NR | M | 6 | Liver, aorta, heart, spleen, and plasma |
| Rahimi 2004^39^ | USA | C57Bl/6 | Jackson | M | 8–12 | Heart, lungs, liver, and spleen. |
| Qin 2003^129^ | USA | C57Bl/6J | Jackson | F | 8 | Brain |
| Methia 2001^139^ | USA | C57Bl/6J | Jackson | M and F | >8 | aortic sinus and aorta |
| Denis 2001^42^ | USA | C57BL/6J | Jackson | F | 8 | Mesentery, brain, skin, lung |
| Andre 2000^40^ | USA | C57BL/6J/129Sv | NR | M and F | NR | Mesenteric venules |
| Wasowska 2001^41^ | USA | C57Bl/6 | Jackson | M | 8-12 | Heart |
| Denis 2001^121^ | USA | C57BL/6J/129Sv | NR | M | NR | Venules, Endothelium, and Leukocytes |
| VanKleef 2000^131^ | Netherlands | NR | NR | M and F | 12-14 | Nephrons and Kidney Tubules |
| Terraube 2007^43^ | France | C57Bl/6 | NR | NR | 6-10 | lungs |
| Qian 2021^44^ | USA | C57Bl/6J | NR | M and F | 2 to 3 months | Liver |
| Liu 2012^140^ | USA | C57Bl/6 | Jackson | M and F | >8 | Liver, kidney, and heart. |
| Jiang 2024^81^ | China | C57Bl/6J | Shulaibao Biotechnology | M | 6-8 | Brain |
| Mang 2024^130^ | China | C57Bl/6N | Vital River | M | 8-10 | Heart |
| South 2024^45^ | UK | C57Bl/6J | NR | NR | NR | Brain |
| Li 2024^82^ | USA | C57BL/6 and BALB/c | Jackson | F | 6–8 | Liver, Small intestine, and lungs. |
| Liu 2024^113^ | China | C57Bl/6 | GemPharmatech | M | 6–8 | Lungs |
| Xie 2024^96^ | China | C57Bl/6 | NR | M | 8 | NR |
| Schellenberg 2024^166^ | France | C57Bl/6N | Charles River | M | 8-10 | Colon |
| Zifkos 2024^84^ | Germany | C57Bl/6J | NR | M | NR | Lungs |
| Jin 2024^126^ | China | C57Bl/6J | Chubu Kagaku Shizai | M | 7 | Brain |
| Ozawa 2024^158^ | USA | NR | NR | M and F | 20–25 | Aorta |
| Sharma 2024^114^ | Canada | C57Bl/6 | Charles River | M and F | 3 to 12 months | Lung, liver, and kidneys |
| Saeki 2023^59^ | Japan | CD1 | Charles River | F | 6-8 | Liver |
| Kim 2023^46^ | USA | C57Bl/6J | Jackson | M and F | 8 to 9 | Lungs |
| Oleshko 2023^93^ | Germany | NR | in-house | NR | 8-12 | Spleen and bone marrow |
| Xu 2024^83^ | China | C57Bl/6J | Vital River | M | 6-8 | Intestines |
| Onodera 2023^47^ | Japan | C57Bl/6 | Jackson | NR | 8-12 | Lungs |
| Gao 2023^48^ | USA | C57Bl/6J | Jackson | M and F | 8-12 | Liver |
| Rossato 2023^49^ | Italy | Townes Transgenic Mice | Jackson and Charles | M | 12-16 | NR |
| Ozawa 2023^60^ | USA | C57Bl/6J | Jackson | M and F | 10-20 | Heart |
| An 2023^94^ | USA | HbSS-Townes129/B6 | In-house | M and F | 11–14 | Microvasculature |
| Lin 2022^167^ | China | C57Bl/6 | Shanghai SLAC | M | 7–8 | Liver |
| Choi 2023^50^ | Canada | C57Bl/6 | Jackson | M | 8-10 | Inferior vena cava and thrombi |
| DeWilde 2023^85^ | Belgium | C57Bl/6J | NR | M and F | 8-12 | Brain |
| Ozawa 2022^141^ | USA | C57Bl/6 | Jackson | NR | 14-50 | Heart |
| Fukui 2022^122^ | USA | C57Bl/6J and DBA/1 J | Jackson | M | 8-12 | Synovial tissue and joints |
| Shi 2022^148^ | USA | Townes mice | Jackson | NR | NR | Liver, kidney, lung, heart, and brain. |
| Rossato 2022^86^ | Austria | Townes mice | Jackson | M and F | 3 to 4 months | Lungs and kidneys |
| Courson 2022^51^ | USA | C57Bl/6J and C57BL/6 | Jackson | M | 12-20 | Cremaster muscle |
| Stivala 2022^145^ | Switzerland | HbS, Berkeley mice | Jackson | M | 8-12 | Aorta, lungs, liver, and kidneys |
| Guo 2022^146^ | China | C57Bl/6 | Vital River | M | 6 | Abdominal aorta, abdominal skin, and mesentery |
| Yu 2022^142^ | China | C57Bl/6J | Model Animal Research Center | M | 8 | Aorta |
| South 2022^57^ | UK | C57Bl/6 | Jackson | M | 10-13 | Plasma, Striatum and cortex |
| Zhou 2022^87^ | China | C57Bl/6 | Shanghai SLAC | M | 12-20 | kidneys |
| Meng 2021^52^ | China | C57Bl/6 | Cavens | NR | 8–12 | kidneys |
| Heger 2024^88^ | USA | C57Bl/6J | Jackson | M | NR | heart |
| Xu 2023^123^ | China | C57Bl/6J | Harvard | M | 6 | Brain |
| Macarthur 2024^89^ | USA | NR | Jackson | M | 8-12 | Lungs |
| Zelaya 2023^53^ | Japan | BALB/c | NR | NR | 6 | Lungs and blood |
| Li 2022^124^ | China | C57/BL6N | Beijing Vital River | M | 8 | Lungs |
| Tan 2016^115^ | China | C57Bl/6 | Cyagen Biosciences | M | 6–8 | Lungs |
| Schoner 2015^149^ | USA | CD-1 | Charles River | M and F | 10-24 | Heart and Liver |
| Emmerechts 2012^170^ | Belgium | C57Bl/6J | Janvier | M | 10-80 | Lungs |
| Dmitrieva 2014^147^ | USA | 129S6, 129SVE; | Taconic | NR | 3 months | Liver, lungs, kidney, and blood |

*NR* not reported, *USA* United States of America, *UK* United Kingdom, *KO* Knockout

**Supplementary Table S2. Inflammation model characteristics**

| **Author** | **Method of Inflammation** | **Sterile vs infectious** | **Inflammatory Trigger** | **Induction method** | **Route** | **Analgesia** | **Key outcome measures** |
| --- | --- | --- | --- | --- | --- | --- | --- |
| Wohner 2021^21^ | Acute | Sterile | phorbol-12-myristate-13-acetate (PMA) (for endothelial stimulation) and ristocetin (to enhance VWF activation). | Chemical | IV | NR | Biomarkers |
| Meng 2021^22^ | Acute | Sterile | cisplatin (CP) administration (20 mg/kg) to induce acute kidney injury (AKI) | Chemical | IV | NR | Biomarkers, histology |
| Xiao 2021^23^ | Acute | Sterile | Short-term LPS exposure (2–24 hours) in mice. | Chemical | IP | NR | Biomarkers, histology |
| Nguyen 2021^24^ | Acute | Sterile | IVIg (and recombinant IL-11 in mice) | Chemical | IP | NR | Biomarkers |
| Xiao 2020^25^ | Acute | Sterile | LPS (5 mg/kg) to induce endotoxemia | Chemical | IP | NR | Biomarkers, histology, survival |
| Groeneveld 2020^26^ | Acute | Sterile | Acetaminophen (APAP) overdose to induce acute liver injury | Chemical | IP | NR | Biomarkers, histology, survival |
| Chen 2019^27^ | Acute | Sterile | Acute pulmonary embolism (APE) induced by a chemical mixture of collagen and epinephrine | Chemical | IV | Yes | Biomarkers, histology, survival |
| Sorvillo 2019^28^ | Acute | Sterile | PAD4 (peptidylarginine deiminase type IV) | Chemical | IV | Yes | Biomarkers, histology, survival |
| Sun 2017^29^ | Acute | Sterile | Carbon tetrachloride (CCl₄) | Chemical | IP | NR | Biomarkers, histology |
| Ayme 2017^30^ | Acute | Sterile | Immune complexes (ICV) and chemical irritant (ICD) | Chemical | IV | Yes | Biomarkers, histology |
| Zitomersky 2017^31^ | Acute | Sterile | Dextran Sodium Sulfate (DSS) induced colitis | Chemical | Oral | Yes | Biomarkers, histology |
| Zhang 2016^32^ | Acute | Sterile | Lipopolysaccharide (LPS) and exogenous calf thymus histones (CTH) | Chemical | IV | Yes | Biomarkers, histology |
| Hilgruber 2014^33^ | Acute | Sterile | Immune complexes (ICs) in a reverse passive Arthus reaction model to mimic immune complex–mediated vasculitis (ICV) | Chemical | IP | NR | Biomarkers, histology |
| JayakumarAmirtharaj 2014^34^ | Acute | Sterile | Dimethylnitrosamine (DMN) to induce chemical hepatotoxicity | Chemical | IP | NR | Biomarkers, histology |
| Petri 2010^35^ | Acute | Sterile | Thioglycollate to trigger peritonitis and keratinocyte-derived chemokine (KC) to stimulate inflammation in the cremaster muscle | Chemical | IP | Yes | Biomarkers, histology |
| Dieude 2009^36^ | Acute | Sterile | Ferric chloride (FeCl₃) applied to the carotid artery in mice to create a localized vascular injury | Chemical | Topical | Yes | Biomarkers, histology |
| Noubade 2008^37^ | Acute | Sterile | Pertussis toxin (PTX) as an adjuvant in the experimental allergic encephalomyelitis (EAE) model, mimicking multiple sclerosis | Chemical | IV | NR | Biomarkers, histology |
| Mimuro 2008^38^ | Acute | Sterile | lipopolysaccharide (LPS) injection to create endotoxemia | Chemical | IP | NR | Biomarkers |
| Rahimi 2004^39^ | Acute | Sterile | Alloantibodies (AlloAbs) targeting donor MHC antigens in a mouse cardiac transplantation model | Chemical | IV | Yes | Biomarkers, histology |
| Andre 2000^40^ | Acute | Sterile | Calcium ionophore A23187 (a Weibel-Palade body secretagogue) | Chemical | IP | Yes | Biomarkers, histology |
| Wasowska 2001^41^ | Acute | Sterile | Passive transfer of monoclonal alloantibodies (mAbs) specific for donor MHC class I antigens | Chemical | IV | NR | Biomarkers, histology |
| Denis 2001^42^ | Acute | Sterile | Calcium ionophore A23187 (a Weibel–Palade body secretagogue) applied to mesenteric venules | Chemical | Lumbar injection | NR | Biomarkers, histology |
| Terraube 2007^43^ | Acute | Sterile | Tumor metastasis (via IV injection of B16-BL6 or LLC cells) | Chemical | IV | NR | Biomarkers, histology, survival |
| Qian 2021^44^ | Acute | Sterile | Acetaminophen (APAP) to induce liver injury | Chemical | IP | NR | Biomarkers, Histology, Survival |
| South 2024^45^ | Acute | Sterile | Interleukin-1β (IL-1β) stimulation of an hCMEC/D3 endothelial monolayer | Chemical | IP | NR | Biomarkers, Histology |
| Kim 2023^46^ | Acute | Sterile | Recombinant SARS-CoV-2 spike protein S1 domain (SP-S1). | Chemical | Intranasal | NR | Biomarkers, Histology, Survival |
| Onodera 2023^47^ | Acute | Sterile | lipopolysaccharide (LPS) to create a lung injury and inflammation model | Chemical | Intratracheal | Yes | Biomarkers, Histology, Survival |
| Gao 2023^48^ | Acute | Sterile | Endotoxemia induced by lipopolysaccharide (LPS) | Chemical | IP | NR | Biomarkers, Histology, Survival |
| Rossato 2023^49^ | Acute | Sterile | Hypoxia/reoxygenation-induced pathologies in a mouse model of human sickle cell disease | Chemical | IV | NR | Biomarkers, survival |
| Choi 2023^50^ | Acute | Sterile | LPS-induced inflammation was combined with IVC stenosis to study venous thrombosis and thrombus composition. | Chemical | IP | Yes | Biomarkers, Histology |
| Courson 2022^51^ | Acute | Sterile | Acute inflammation induced by administration of calf thymus histones (containing H1, H2A, H2B, H3, and H4) | Chemical | IV | Yes | Biomarkers, Histology |
| Meng 2021^52^ | Acute | Sterile | Cisplatin-induced acute kidney injury (AKI) model | Chemical | IV | NR | Biomarkers, Histology, Survival |
| Zelaya 2023^53^ | Acute | Sterile | Administration of peptidoglycan from L. rhamnosus (PG-Lr1505) followed by poly(I:C) | Chemical | Nasal | Yes | Biomarkers, Histology |
| Tahir 2020^54^ | Acute | Sterile | CD40L (stimulating endothelial CD40). | Chemical | Local superfusion | Yes | Biomarkers, histology |
| Latifi 2019^55^ | Acute | Sterile | Ponatinib (drug-induced endothelial injury and oxidative stress) | Chemical (Ponatinib treatment) | Oral | Yes | Biomarkers, histology, survival |
| Bonnefoy 2006^56^ | Acute | Sterile | Calcium ionophore A23187 to stimulate endothelial cell release | Chemical | IV | Yes | Biomarkers, Histology, and survival |
| South 2022^57^ | Acute | Sterile | Ischemia–reperfusion injury induced by Transient MCAo (tMCAo) combined with systemic inflammation (IL-1β injection) | Chemical | IP | NR | Biomarkers, Histology, survival |
| Patel 2008^58^ | Acute | Sterile | administration of LPS to induce endotoxemia | Chemical | IP | Yes | Biomarkers and histology |
| Saeki 2023^59^ | Acute | Sterile | Monocrotaline (MCT) to induce sinusoidal obstruction syndrome (SOS) | Chemical | IP | NR | Biomarkers |
| Ozawa 2023^60^ | Acute | Sterile | myocardial ischemia-reperfusion injury by temporarily occluding the left anterior descending (LAD) coronary artery | Physical | Surgical | Yes | Biomarkers, Histology, Survival |
| Plautz 2021^61^ | Acute | Sterile | Severe traumatic injury and hemorrhagic shock | Physical | IP | Yes | Biomarkers, histology, survival |
| Xu 2021^62^ | Acute | Sterile | Desmopressin and histamine (to stimulate VWF release). | Physical | Surgical | Yes | Biomarkers |
| Shentu 2021^63^ | Acute | Sterile | ischemic injury (myocardial infarction) | Physical | Surgical | Yes | Biomarkers, histology |
| Cui 2021^64^ | Acute | Sterile | traumatic brain injury (TBI)-induced coagulopathy | Physical | Surgical | Yes | Biomarkers, histology, survival |
| Wong 2020^65^ | Acute | Sterile | Skin allograft transplantation (alloimmune trigger) | Physical | Surgical | Yes | Biomarkers, histology, survival |
| Zhu 2020^66^ | Acute | Sterile | Disturbed blood flow induced by ligation of the left external carotid artery (LECA) | Physical | Surgical | Yes | Biomarkers, histology |
| Denorme 2021^67^ | Acute | Sterile | Ischemic stroke induced by transient middle cerebral artery occlusion (MCAO) | Physical | Surgical | Yes | Biomarkers, histology, survival |
| Ono 2019^68^ | Acute | Sterile | Surgical ischemia-reperfusion injury | Physical | Surgical | Yes | Biomarkers, histology, survival |
| Horioka 2019^69^ | Acute | Sterile | Physical stress (hypothermia and rewarming) | Physical | Environmental | Yes | Biomarkers, histology, survival |
| Zhou 2019^70^ | Acute | Sterile | Renal ischemia-reperfusion (I/R) injury | Physical | Surgical | Yes | Biomarkers, histology, survival |
| Wu 2018^71^ | Acute | Sterile | Traumatic brain injury (TBI) induced by fluid percussion injury (FPI) | Physical | Surgical | Yes | Biomarkers, histology, survival |
| Moccetti 2018^72^ | Acute | Sterile | Closed-chest ligation of the left anterior descending (LAD) coronary artery | Physical | Surgical | Yes | Biomarkers, histology, survival |
| Nicolay 2018^73^ | Acute | Sterile | Cellular stress leading to eryptosis | Physical | Surgical | Yes | Biomarkers, histology |
| Urisono 2018^74^ | Acute | Sterile | Hepatic ischemia–reperfusion (I/R) injury | Physical | Surgical | Yes | Biomarkers, histology |
| Kim 2017^75^ | Acute | Sterile | Renal ischemia–reperfusion injury (IRI) | Physical | Surgical | NR | Biomarkers, histology |
| Zhu 2016^76^ | Acute | Sterile | Intracerebral haemorrhage (ICH) via injury | Physical | Surgical | Yes | Biomarkers, histology |
| Cai 2015^77^ | Acute | Sterile | Intracerebral haemorrhage (ICH) via injury | Physical | Surgical | NR | Biomarkers, Histology, Survival |
| Savchenko 2014^78^ | Acute | Sterile | Myocardial ischemia/reperfusion (MI/R) using a 24-hour surgical MI/R mouse model | Physical | Surgical | Yes | Biomarkers, histology, survival |
| Gandhi 2012 - 1^79^ | Acute | Sterile | Myocardial ischemia/reperfusion (I/R) using a left anterior descending (LAD) coronary artery ligation model | Physical | Surgical | Yes | Biomarkers, histology |
| De Meyer 2012^80^ | Acute | Sterile | Myocardial ischemia/reperfusion (MI/R) using a left anterior descending (LAD) coronary artery occlusion model | Physical | Surgical | Yes | Biomarkers, histology, survival |
| Jiang 2024^81^ | Acute | Sterile | Middle cerebral artery occlusion (MCAO) to induce ischemic brain injury | Physical | Surgical | Yes | Biomarkers, Histology, Survival |
| Li 2024^82^ | Acute | Sterile | Graft-versus-host disease (GVHD) in mice, triggered by allogeneic hematopoietic stem cell transplantation (allo-HSCT) | Physical | Surgical | NR | Biomarkers, Histology, Survival |
| Xu 2024^83^ | Acute | Sterile | Total abdominal irradiation | Physical | Radiation | Yes | Biomarkers, Histology, Survival |
| Zifkos 2024^84^ | Acute | Sterile | Inferior vena cava (IVC) ligation (stenosis) | Physical | Surgical | NR | Biomarkers, Histology, Survival |
| DeWilde 2023^85^ | Acute | Sterile | Transient middle cerebral artery occlusion (MCAO) was used to induce ischemia-reperfusion injury and inflammation in the brain. | Physical | Intracerebral | Yes | Biomarkers, Histology |
| Rossato 2022^86^ | Acute | Sterile | Hypoxia/reoxygenation (H/R) exposure in sickle cell mice to mimic vaso-occlusive crisis. | Physical | Environmental | NR | Biomarkers, Histology |
| Zhou 2022^87^ | Acute | Sterile | Ischemia/reperfusion (IR) model causing acute kidney injury (AKI) | Physical | Surgical | Yes | Biomarkers, Histology, Survival |
| Heger 2024^88^ | Acute | Sterile | Acute myocardial infarction (MI) induced by permanent ligation of the left anterior descending (LAD) coronary artery | Physical | Surgical | Yes | Biomarkers, Histology, Survival |
| Macarthur 2024^89^ | Acute | Sterile | Polytrauma model including femur fracture, gastrocnemius crush, and laparotomy | Physical | Surgical | Yes | Biomarkers, Histology, Survival |
| Fujioka 2012^90^ | Acute | Sterile | Ischemia–reperfusion injury induced by 30-minute middle cerebral artery occlusion | Physical | Surgical | Yes | Biomarkers, Histology, survival |
| Fujioka 2010^91^ | Acute | Sterile | Transient middle cerebral artery occlusion for 30 minutes | Physical | Surgical | NR | Biomarkers, Histology, survival |
| Bayat 2015^92^ | Acute | Sterile | Anti–HNA-3a alloantibodies (antibodies against human neutrophil antigen-3a) | Genetic | N/A | NR | Biomarkers, Histology |
| Oleshko 2023^93^ | Acute | Sterile | Recombinant human factor VIII (rhFVIII) in hemophilia A mice | Genetic | N/A | NR | Biomarkers, Histology |
| An 2023^94^ | Acute | Sterile | Sickle red blood cell–derived extracellular vesicles (SS REVs) generated from HbSS RBCs | Genetic | N/A | NR | Biomarkers, Histology, Survival |
| Chauhan 2008^95^ | Acute | Sterile | Genetic deficiency of ADAMTS13 | Genetic | N/A | Yes | Biomarkers and Histology |
| Xie 2024^96^ | Acute | Infectious | Lipopolysaccharide (LPS), administered intraperitoneally at 50 mg/kg to create a sepsis model | Bacterial | IP | NR | Biomarkers, Histology, Survival |
| Na 2020^97^ | Acute | Infectious | Live *Staphylococcus aureus* bacteria to induce septic arthritis | Bacterial | IV | NR | Biomarkers, histology |
| Peetermans 2020^98^ | Acute | Infectious | Live *Staphylococcus aureus* bacteria (Newman strain) to induce sepsis | Bacterial | IV | NR | Biomarkers, histology, survival |
| Liesenborghs 2016^99^ | Acute | Infectious | *Staphylococcus lugdunensis* to study adhesion and endocarditis development. | Bacterial | IV | Yes | Biomarkers, Histology, Survival |
| Claes 2014^100^ | Acute | Infectious | *Staphylococcus aureus* to study adhesion and microthrombus formation. | Bacterial | IV | Yes | Biomarkers |
| Pappelbaum 2013^101^ | Acute | Infectious | *Staphylococcus aureus* to study adhesion under flow and in vivo. | Bacterial | IV | NR | Biomarkers, histology, survival |
| Huang 2010^102^ | Acute | Infectious | Shiga toxin B subunits (Stx1B and Stx2B), which are components of Shiga toxins produced by *Shigella dysenteriae* and *Escherichia coli* | Bacterial | IV | NR | Biomarkers, histology, survival |
| Liesenborghs 2019^103^ | Acute | Infectious | Live *Staphylococcus aureus* (Newman strain, USA300, or clinical isolates) to study bacterial adhesion and endocarditis development. | Bacterial | IV | Yes | Biomarkers, histology, survival |
| Claes 2017^104^ | Acute | Infectious | *Staphylococcus aureus* strains to study adhesion mechanisms. | Bacterial | IV | Yes | Biomarkers, histology |
| Chung 2008^105^ | Acute | Infectious | Anthrax infection (toxigenic Sterne 34F2 strain in mice) Bacterial infection (Bacillus anthracis) | Bacterial | IP | NR | Histology |
| Chauhan 2008^106^ | Acute | Infectious | Shiga toxin (Stx) to induce thrombotic thrombocytopenic purpura (TTP) in ADAMTS13-deficient mice. | Bacterial | IP | Yes | Histology |
| O'Regan 2016^107^ | Acute | Infectious | Infection with *Plasmodium berghei* ANKA parasites in mice to model experimental cerebral malaria (CM). | Parasitic | IP | Yes | Biomarkers, histology |
| Kraisin 2019^108^ | Acute | Infectious | Infection with *Plasmodium berghei* NK65-E parasites to model malaria-associated acute respiratory distress syndrome (MA-ARDS) | Parasitic | IP | Yes | Biomarkers, histology, survival |
| Kraisin 2020^109^ | Acute | Infectious | Infection with *Plasmodium berghei* ANKA (PbANKA) parasites to model experimental cerebral malaria (ECM) | Parasitic | IP | Yes | Biomarkers, histology, survival |
| Alflen 2017^110^ | Acute | Infectious | Infection with *Aspergillus fumigatus* conidia, creating a model of invasive pulmonary aspergillosis (IPA). | Fungal | Intratracheal | Yes | Biomarkers, histology |
| Patel 2010^111^ | Acute | Infectious | Live gut bacteria (via cecal ligation and puncture ) (infection-induced polymicrobial sepsis) | Polymicrobial | IP | Yes | Biomarkers, survival |
| Lerolle 2009^112^ | Acute | Infectious | Live gut bacteria introduced via CLP (infection-induced polymicrobial sepsis) | Polymicrobial | IP | NR | Biomarkers, survival |
| Liu 2024^113^ | Acute | Infectious | Induced by live gut bacteria via cecal ligation and puncture (CLP) in mice, which creates polymicrobial sepsis | Polymicrobial | IP | Yes | Biomarkers, Histology, Survival |
| Sharma 2024^114^ | Acute | Infectious | Induced peritonitis (FIP), which involves intraperitoneal injection of rat fecal slurry into mice (polymicrobial abdominal sepsis). | Polymicrobial | IP | Yes | Biomarkers, Histology, Survival |
| Tan 2016^115^ | Acute | Infectious | Cecal ligation and puncture (CLP) polymicrobial sepsis model | Polymicrobial | IP | Yes | Histology |
| Jin 2020^116^ | Chronic | Sterile | Chronic psychological stress (CPS) via 2-week immobilization stress protocol (4 hours/day). | Chemical | Topical | Yes | Biomarkers, histology |
| Lu 2020^117^ | Chronic | Sterile | Myelin oligodendrocyte glycoprotein (MOG₃₅–₅₅) peptide immunization to trigger experimental autoimmune encephalomyelitis (EAE**)** | Chemical | IP | Yes | Biomarkers, histology |
| Suraj 2019^118^ | Chronic | Sterile | Tumor cells (4T1 orthotopic implantation). | Chemical | IP | Yes | Biomarkers, histology |
| Dhanesha 2017^119^ | Chronic | Sterile | Streptozotocin (STZ) to create type 1 diabetes | Chemical | Intraperitoneal (i.p.) | Yes | Biomarkers, histology |
| Adam 2016^120^ | Chronic | Sterile | Reverse passive Arthus (rpA) reaction induced by injection of BSA followed by anti-BSA IgG. | Chemical | Intraperitoneal (i.p.) | Yes | Biomarkers, histology |
| Denis 2001^121^ | Chronic | Sterile | Recombinant human interleukin-11 (rhIL-11). | Chemical | intravenous (IV) | NR | Biomarkers |
| Fukui 2022^122^ | Chronic | Sterile | Type II collagen + CFA (autoimmune trigger) | Chemical | Subcutaneous (SC) | NR | Biomarkers, histology |
| Xu 2023^123^ | Chronic | Sterile | FeCl₃ (chemical injury) + chronic restraint stress | Chemical | topical | NR | Biomarkers, histology |
| Li 2022^124^ | Chronic | Sterile | Bleomycin (chemical inducer of systemic sclerosis) | Chemical | Intragastric | Yes | Biomarkers, histology |
| Xiang 2015^125^ | Chronic | Sterile | Hyperglycemia in diabetes mellitus. | Chemical | IP | NR | Biomarkers, Histology, Survival |
| Jin 2024^126^ | Chronic | Sterile | Chronic psychological stress (CPS) with FeCl₃-induced carotid artery injury | Chemical | IP | Yes | Biomarkers, Histology, Survival |
| Zhou 2021^127^ | Chronic | Sterile | Renal ischaemia/reperfusion (IR) injury to induce chronic kidney disease (CKD). | Physical | surgical | Yes | Biomarkers, Histology |
| Witsch 2018^128^ | Chronic | Sterile | Mechanical stress via Ascending Aortic Constriction (AAC) and Angiotensin II (AngII) infusion | Physical | Surgical | Yes | Biomarkers, histology |
| Qin 2003^129^ | Chronic | Sterile | Mechanical low shear stress induced by carotid artery ligation | Physical | surgical | Yes | Biomarkers, histology |
| Mang 2024^130^ | Chronic | Sterile | Pressure overload (transverse aortic constriction, TAC) and myocardial infarction (MI) in mouse models of heart failure. | Physical | Surgical | Yes | Biomarkers |
| VanKleef 2000^131^ | Chronic | Sterile | Ionizing radiation (fractionated doses: 20 × 1.0 Gy or 10 × 1.6 Gy ± re-irradiation) | Physical | Radiation | NR | Biomarkers, Histology |
| Ozawa 2020^132^ | Chronic | Sterile | Atherosclerosis in LDL-R⁻/⁻ and LDL-R⁻/⁻ADAMTS13⁻/⁻ mice fed a Western-style diet (WSD) | Diet | Oral (diet) | Yes | Biomarkers, histology |
| Michels 2020^133^ | Chronic | Sterile | High-fat diet to create diet-induced obesity (DIO), which triggers chronic low-grade inflammation | Dietary | Oral (diet) | Yes | Biomarkers, histology |
| Yang 2020^134^ | Chronic | Sterile | High-fat diet to create diet-induced obesity (DIO) | Dietary | Oral | NR | Biomarkers, histology |
| Doddapattar 2018^135^ | Chronic | Sterile | Iigh-fat “Western” diet for 14 weeks in Apoe-deficient mice to induce atherosclerosis and vascular inflammation. | Dietary | Oral | Yes | Biomarkers, histology |
| Geys 2017^136^ | Chronic | Sterile | High-fat diet for 15 weeks to create diet-induced obesity and liver steatosis (NASH model). | Diet | Oral | NR | Biomarkers, histology |
| Hung 2015^137^ | Chronic | Sterile | Streptozotocin to create diabetes in mice. | Diet | Oral | NR | Biomarkers, histology |
| Iwaki 2006^138^ | Chronic | Sterile | LDL cholesterol-driven atherosclerosis in predisposed mice. | Diet | Oral | NR | Biomarkers, histology |
| Methia 2001^139^ | Chronic | Sterile | atherogenic diet rich in saturated fat and cholesterol to induce atherosclerosis | Diet | Oral | NR | Biomarkers, histology |
| Liu 2012^140^ | Chronic | Sterile | Western-type high-fat diet (HF), which induced obesity in wild-type mice and severe hypercholesterolemia with early atherosclerosis in apoE knockout mice. | Diet | Oral | NR | Biomarkers |
| Ozawa 2022^141^ | Chronic | Sterile | Genetic deficiency of ADAMTS13 in hyperlipidemic mice (*LDLR⁻/⁻ ADAMTS13⁻/⁻*) fed a Western-style diet. | Diet | Oral | NR | Biomarkers, histology, |
| Yu 2022^142^ | Chronic | Sterile | High-fat diet (HFD) in ApoE⁻/⁻ mice to model atherosclerosis | Diet | Oral | NR | Biomarkers, histology |
| Jin 2012^143^ | Chronic | Sterile | Atherosclerosis induced by a high-fat Western diet in genetically modified mice (ApoE⁻/⁻ and Adamts13⁻/⁻ ApoE⁻/⁻). | Diet | Oral | Yes | Biomarkers, histology |
| Gandhi 2012-2^144^ | Chronic | Sterile | High-fat Western diet in genetically modified mice (ApoE⁻/⁻ and Adamts13⁻/⁻ ApoE⁻/⁻). | Diet | Oral | Yes | Biomarkers, histology |
| Stivala 2022^145^ | Chronic | Sterile | Genetic sickle cell disease model (Berkeley mice) + dietary ALA intervention | Dietary | Oral (dietary ALA) | NR | Biomarkers, histology |
| Guo 2022^146^ | Chronic | Sterile | Diet-induced atherosclerosis in ApoE⁻/⁻ mice fed a hypercholesterolemic diet (HCD) | Dietary | Oral | NR | Biomarkers, histology |
| Dmitrieva 2014^147^ | Chronic | Sterile | Hypernatremia / hypertonic stress by dehydration, high salt intake, or hypertonic saline therapy | Diet | Oral | NR | Biomarkers, Histology |
| Shi 2022^148^ | Chronic | Sterile | TNF challenge or hypoxia/reoxygenation stress in sickle cell mice | Genetic | N/A | Yes | Biomarkers, Histology |
| Schoner 2015^149^ | Chronic | Sterile | Heart failure-induced endothelial dysfunction (Genetic heart-failure model) | Genetic | N/A | NR | Biomarkers, Histology |
| Kiouptsi 2017^150^ | Chronic | Sterile | Genetically induced by Factor VIII (F8) deficiency in mice (a hemophilia A model) | Genetic | NA | NR | Biomarkers, histology |
| Chen 2017^151^ | Chronic | Sterile | Repeated intravenous immunization with recombinant human factor VIII (rhF8) | Genetic | NA | Yes | Biomarkers, histology |
| Shim 2015^152^ | Chronic | Sterile | Genetic hyperlipidemia and atherosclerosis in mice (LDL receptor and Apobec-1 double knockout). | Genetic | NA | NR | Biomarkers, histology |
| Rhieu 2014^153^ | Chronic | Sterile | Effects of vWF deletion on hematopoiesis and pulmonary fibrosis. | Genetic | N/A | NR | Biomarkers, histology |
| Delignat 2012^154^ | Chronic | Sterile | repeated intravenous administration of recombinant human factor VIII (FVIII) in FVIII-deficient mice | Genetic | NA | Not specified | Biomarkers, histology |
| McCarty 2010^155^ | Chronic | Sterile | Genetic hyperlipidemia (LDLR⁻/⁻ ApoBec-1⁻/⁻ mice) | Genetic | NA | NR | Biomarkers, histology |
| Delignat 2007^156^ | Chronic | Sterile | FVIII-deficient mice (hemophilia A model). | Genetic | NA | NR | Biomarkers |
| Kallas 2007^157^ | Chronic | Sterile | FVIII-deficient mice (hemophilia A model) | Genetic | NA | NR | Biomarkers |
| Ozawa 2024^158^ | Chronic | Sterile | Myocardial infarction (MI) induced by transient left anterior descending (LAD) coronary artery occlusion in hyperlipidemic mice (*LDLR⁻/⁻ Apobec1⁻/⁻*). | Genetic | NA | Yes | Biomarkers, histology |
| Bauer 2015^159^ | Chronic | Sterile | Tumor microenvironment | Genetic | N/A | NR | Biomarkers, Histology, Survival |
| EspiritoSanto 2004^160^ | Chronic | Sterile | Mice with inducible hepatic LRP deficiency on an LDLR–/– APOE–/– background | Genetic | IP | NR | Biomarkers, Histology, and survival |
| Kang 2019^161^ | Chronic | Sterile | Genetic deficiency of α‑galactosidase A (GLA) to model Fabry disease | Genetic | NA | NR | Biomarkers |
| Mojiri 2013^162^ | Chronic | Sterile | Chronic hypoxia (progressively reduced oxygen for 35 days) to induce pulmonary hypertension | Environmental | Hypoxia Chamber | NR | Biomarkers, Histology |
| Dincel 2016^163^ | Chronic | Infectious | Infection with *Toxoplasma gondii* (ME49 strain) | Parasitic | IP | Yes | Biomarkers, histology |
| Poole 2021^164^ | Acute and Chronic | Sterile | α‑naphthylisothiocyanate (ANIT) induced bile‑duct injury, necrosis and an inflammatory response, allowing the impact of VWF deficiency on cholestatic liver injury to be examined | Chemical | Oral and dietary | NR | Biomarkers, histology |
| Joshi 2017^165^ | Acute and Chronic | Sterile | Liver injury and fibrosis induced by CCl4 | Chemical (CCl4) | IP | NR | Biomarkers, histology |
| Schellenberg 2024^166^ | Acute and Chronic | Sterile | Dextran sulfate sodium (DSS) in mice to model colitis. | Chemical | Oral | Yes | Biomarkers, histology |
| Lin 2022^167^ | Acute and Chronic | Sterile | LPS + D-GalN (with CCl₄ pre-treatment for acute-on-chronic liver failure ). | Chemical | IP and oral for MHLQD | NR | Biomarkers, histology, survival |
| Michels 2016^168^ | Acute and chronic | Sterile | Histones (including unfractionated histones and lysine-rich or arginine-rich histone fractions) to induce inflammatory responses | Chemical | IP | NR | Biomarkers |
| Ostertag 2016^169^ | Acute and chronic | Sterile | Human anti-ADAMTS13 autoantibody fragments (scFv) | Chemical | IP | Yes | Biomarkers, Histology, Survival |
| Emmerechts 2012^170^ | Subchronic | Sterile | Traffic-related air pollution (particulate matter, PM in tunnel air) | Environmental | Inhalation | NR | Biomarkers |

**Supplementary Table S3. Inflammation outcomes pertaining to VWF and VWF-mediated processes**

| **Author** | **↑ VWF Levels** | **↑ VWF Activity** | **VWF Multimer Distribution** | **Platelet Adhesion** | **Endothelial Dysfunction** | **Inflammatory Markers** | **VWF-Related Proteins** | **Therapeutic potential of ADAMTS13** | **Leukocyte recruitment** | **Neutrophil Infiltration** | **Platelet aggregation** |
| --- | --- | --- | --- | --- | --- | --- | --- | --- | --- | --- | --- |
| Plautz 2021^61^ | Yes | Yes | Yes | NR | NR | NR | Yes | NR | NR | NR | NR |
| Wohner 2021^21^ | Yes | NR | NR | NR | NR | NR | NR | NR | NR | NR | NR |
| Xu 2021^62^ | Yes | NR | Yes | Yes | NR | NR | Yes | NR | NR | NR | Yes |
| Meng 2021^22^ | NR | NR | NR | NR | NR | Yes | Yes | Yes | NR | NR | NR |
| Xiao 2021^23^ | Yes | NR | NR | NR | Yes | NR | NR | NR | NR | NR | NR |
| Nguyen 2021^24^ | Yes | NR | NR | NR | NR | NR | Yes | NR | NR | NR | NR |
| Poole 2021^164^ | Yes | Yes | NR | Yes | NR | NR | NR | NR | NR | Yes | Yes |
| Shentu 2021^63^ | Yes | Yes | NR | Yes | Yes | Yes | NR | NR | NR | NR | NR |
| Na 2020^97^ | NR | NR | NR | NR | NR | Yes | NR | NR | NR | NR | NR |
| Cui 2021^64^ | Yes | NR | NR | NR | NR | Yes | Yes | Yes | NR | NR | NR |
| Zhou 2021^127^ | Yes | NR | NR | NR | NR | NR | Yes | Yes | NR | NR | NR |
| Zhu 2020^66^ | Yes | NR | Yes | Yes | Yes | Yes | Yes | NR | NR | NR | Yes |
| Ozawa 2020^132^ | Yes | NR | NR | Yes | Yes | Yes | Yes | Yes | NR | NR | NR |
| Xiao 2020^25^ | Yes | NR | NR | NR | Yes | Yes | Yes | NR | NR | NR | NR |
| Groeneveld 2020^26^ | Yes | Yes | Yes | Yes | NR | NR | Yes | NR | NR | NR | Yes |
| Wong 2020^65^ | NR | NR | NR | NR | Yes | Yes | Yes | Yes | Yes | Yes | NR |
| Peetermans 2020^98^ | NR | NR | Yes | NR | Yes | Yes | Yes | Yes | NR | NR | Yes |
| Denorme 2021^67^ | Yes | NR | NR | Yes | Yes | Yes | NR | NR | Yes | Yes | Yes |
| Jin 2020^116^ | Yes | NR | NR | NR | Yes | Yes | Yes | NR | Yes | Yes | NR |
| Kraisin 2020^109^ | Yes | NR | Yes | NR | Yes | NR | Yes | NR | Yes | NR | Yes |
| Tahir 2020^54^ | Yes | NR | Yes | Yes | Yes | NR | Yes | NR | Yes | NR | Yes |
| Michels 2020^133^ | Yes | NR | NR | Yes | Yes | Yes | Yes | NR | Yes | Yes | Yes |
| Lu 2020^117^ | Yes | NR | Yes | NR | Yes | Yes | Yes | Yes | Yes | Yes | NR |
| Yang 2020^134^ | Yes | NR | NR | NR | NR | Yes. | NR | NR | NR | NR | NR |
| Kang 2019^161^ | Yes | NR | NR | NR | Yes | Yes | NR | NR | NR | NR | NR |
| Liesenborghs 2019^103^ | Yes | NR | NR | Yes | Yes | Yes | NR | NR | Yes | Yes | Yes |
| Latifi 2019^55^ | Yes | Yes | Yes | Yes | Yes | Yes | Yes | Yes | Yes | NR | Yes |
| Ono 2019^68^ | Yes | NR | NR | NR | Yes | Yes | Yes | Yes | Yes | Yes | NR |
| Horioka 2019^69^ | Yes | NR | NR | Yes | NR | Yes | NR | NR | NR | NR | Yes |
| Chen 2019^27^ | Yes | NR | NR | Yes | Yes | Yes | NR | NR | NR | Yes | Yes |
| Sorvillo 2019^28^ | NR | NR | Yes | Yes | NR | Yes | Yes | Yes | NR | Yes | Yes |
| Kraisin 2019^108^ | Yes | NR | Yes | NR | Yes | NR | Yes | NR | NR | NR | NR |
| Suraj 2019^118^ | Yes | NR | NR | NR | Yes | Yes | NR | NR | NR | NR | NR |
| Zhou 2019^70^ | Yes | NR | NR | NR | Yes | Yes | Yes | Yes | Yes | Yes | NR |
| Wu 2018^71^ | Yes | Yes | Yes | Yes | Yes | NR | Yes | Yes | NR | NR | Yes |
| Moccetti 2018^72^ | Yes | NR | NR | NR | Yes | NR | Yes | Yes | Yes | Yes | Yes |
| Nicolay 2018^73^ | Yes | NR | Yes | NR | Yes | NR | NR | Yes | NR | NR | NR |
| Urisono 2018^74^ | NR | NR | Yes | Yes | Yes | NR | Yes | Yes | Yes | Yes | NR |
| Witsch 2018^128^ | Yes | NR | Yes | NR | Yes | Yes | Yes | Yes | Yes | Yes | Yes |
| Doddapattar 2018^135^ | Yes | Yes | Yes | Yes | Yes | Yes | Yes | NR | Yes | Yes | Yes |
| Sun 2017^29^ | Yes | NR | NR | NR | Yes | Yes | NR | NR | NR | NR | NR |
| Kiouptsi 2017^150^ | Yes | Yes | Yes | NR | Yes | Yes | Yes | NR | NR | NR | NR |
| Ayme 2017^30^ | NR | NR | NR | Yes | Yes | NR | NR | NR | Yes | NR | Yes |
| Joshi 2017^165^ | Yes | NR | NR | NR | NR | Yes | NR | NR | NR | NR | NR |
| Dhanesha 2017^119^ | Yes | NR | Yes | Yes | Yes | Yes | Yes | Yes | NR | NR | Yes |
| Claes 2017^104^ | NR | NR | Yes | NR | Yes | NR | Yes | Yes | NR | NR | NR |
| Geys 2017^136^ | Yes | NR | Yes | NR | NR | Yes | Yes | NR | NR | NR | NR |
| Kim 2017^75^ | Yes | NR | Yes | NR | Yes | Yes | Yes | Yes | Yes | Yes | NR |
| Zitomersky 2017^31^ | Yes | NR | NR | NR | NR | Yes | Yes | Yes | Yes | Yes | NR |
| Chen 2017^151^ | NR | NR | NR | NR | NR | Yes. | Yes | NR | NR | NR | NR |
| Alflen 2017^110^ | NR | NR | NR | NR | NR | Yes | Yes | NR | Yes | Yes | NR |
| O'Regan 2016^107^ | Yes | Yes | Yes | NR | Yes. | NR | Yes | NR | NR | NR | NR |
| Zhang 2016^32^ | Yes | NR | NR | NR | Yes | Yes | Yes | NR | Yes | Yes | NR |
| Zhu 2016^76^ | Yes | NR | NR | NR | Yes | Yes | NR | NR | Yes | Yes | NR |
| Michels 2016^168^ | Yes | NR | NR | Yes | Yes | Yes | Yes | NR | NR | NR | Yes |
| Adam 2016^120^ | NR | NR | Yes. | Yes | NR | Yes | NR | NR | Yes | Yes | Yes. |
| Ostertag 2016^169^ | NR | NR | Yes | NR | Yes | NR | Yes | NR | NR | NR | NR |
| Liesenborghs 2016^99^ | NR | NR | NR | Yes | Yes | NR | NR | NR | NR | NR | Yes |
| Dincel 2016^163^ | NR | NR | NR | NR | Yes | Yes | Yes | Yes | Yes | NR | NR |
| Bauer 2015^159^ | Yes | NR | Yes | Yes | Yes | Yes | Yes | Yes | NR | NR | Yes |
| Xiang 2015^125^ | Yes | NR | Yes | Yes | Yes | Yes | Yes | NR | NR | NR | Yes |
| Cai 2015^77^ | NR | NR | NR | NR | Yes | Yes | Yes | Yes | Yes | Yes | NR |
| Hung 2015^137^ | Yes | NR | NR | NR | NR | Yes | Yes | NR | NR | NR | NR |
| Shim 2015^152^ | NR | NR | Yes | Yes | NR | NR | Yes | Yes | NR | NR | NR |
| Bayat 2015^92^ | NR | Yes | NR | NR | NR | Yes | NR | NR | Yes | Yes | NR |
| Rhieu 2014^153^ | NR | NR | NR | NR | NR | NR | NR | NR | Yes | Yes | NR |
| Claes 2014^100^ | NR | Yes | Yes | Yes | NR | NR | Yes | NR | NR | NR | Yes |
| Savchenko 2014^78^ | NR | Yes | NR | NR | NR | Yes | Yes | Yes | Yes | Yes | NR |
| Hilgruber 2014^33^ | Yes | Yes | NR | NR | NR | Yes. | Yes | NR | Yes | Yes | NR |
| Pappelbaum 2013^101^ | NR | Yes | Yes | Yes | NR | NR | Yes | Yes | NR | NR | NR |
| Mojiri 2013^162^ | Yes | Yes | NR | Yes | NR | NR | NR | NR | NR | NR | NR |
| JayakumarAmirtharaj 2014^34^ | NR | NR | NR | NR | Yes | Yes | Yes | NR | NR | NR | NR |
| Gandhi 2012 - 1^79^ | Yes | NR | NR | NR | NR | NR | Yes | Yes | Yes | Yes | NR |
| De Meyer 2012^80^ | Yes | NR | NR | Yes | NR | Yes | Yes | Yes | Yes | Yes | NR |
| Jin 2012^143^ | Yes | NR | Yes | NR | NR | Yes. | Yes | NR | Yes | NR | NR |
| Fujioka 2012^90^ | NR | Yes | Yes | Yes | Yes | Yes | Yes | Yes | Yes | Yes | Yes |
| Gandhi 2012 - 2^144^ | NR | NR | NR | NR | NR | Yes | Yes | NR | Yes | NR | NR |
| Delignat 2012^154^ | Yes | NR | NR | NR | NR | Yes | Yes | NR | NR | NR | NR |
| McCarty 2010^155^ | Yes | Yes | NR | Yes | Yes | NR | Yes | NR | Yes | NR | Yes |
| Huang 2010^102^ | Yes | NR | Yes | Yes | Yes | NR | Yes | NR | NR | NR | Yes |
| Patel 2010^111^ | Yes | NR | NR | Yes | Yes | Yes | NR | NR | NR | NR | NR |
| Petri 2010^35^ | Yes | NR | NR | Yes | Yes | NR | Yes | NR | Yes | Yes | NR |
| Fujioka 2010^91^ | NR | Yes | Yes | Yes | Yes | Yes | Yes | Yes | Yes | Yes | Yes |
| Dieude 2009^36^ | Yes | NR | NR | NR | Yes | Yes | Yes | NR | Yes | Yes | NR |
| Chauhan 2008^95^ | Yes | NR | Yes | Yes | Yes | Yes | NR | NR | Yes | Yes | NR |
| Noubade 2008^37^ | NR | NR | NR | NR | Yes | Yes | NR | NR | Yes | Yes | NR |
| Chauhan 2008^106^ | NR | Yes | NR | Yes | NR | NR | NR | NR | NR | NR | NR |
| Chung 2008^105^ | Yes | No | Yes | Yes | Yes | NR | Yes | NR | NR | NR | No |
| Lerolle 2009^112^ | Yes | Yes | Yes | NR | Yes | Yes | Yes | Yes | Yes | NR | NR |
| Patel 2008^58^ | Yes | Yes | NR | Yes | Yes | NR | Yes | NR | NR | NR | Yes |
| Mimuro 2008^38^ | Yes | Yes | Yes | NR | Yes | Yes | Yes | NR | NR | NR | NR |
| Iwaki 2006^138^ | Yes | Yes | NR | Yes | Yes | NR | Yes | NR | Yes | NR | Yes |
| Bonnefoy 2006^56^ | Yes | Yes | Yes | Yes | Yes | NR | NR | NR | NR | NR | Yes |
| Delignat 2007^156^ | Yes | NR | NR | NR | NR | NR | Yes | NR | NR | NR | NR |
| Kallas 2007^157^ | Yes | Yes | Yes | NR | NR | NR | Yes | NR | NR | NR | NR |
| EspiritoSanto 2004^160^ | Yes | NR | NR | NR | Yes | NR | Yes | NR | NR | NR | NR |
| Rahimi 2004^39^ | Yes | Yes | NR | Yes | Yes | Yes | NR | NR | Yes | NR | Yes |
| Qin 2003^129^ | Yes | NR | Yes | NR | Yes | NR | NR | NR | NR | NR | NR |
| Methia 2001^139^ | NR | NR | NR | Yes | Yes | Yes | Yes | NR | Yes | NR | Yes |
| Denis 2001^42^ | Yes | Yes | Yes | NR | NR | NR | Yes | NR | NR | NR | NR |
| Andre 2000^40^ | Yes | Yes | NR | Yes | No | NR | Yes | NR | Yes | NR | No |
| Wasowska 2001^41^ | Yes | Yes | NR | Yes | Yes | Yes | Yes | NR | NR | NR | Yes |
| Denis 2001^121^ | No | NA | NR | NR | Yes | NR | Yes | NR | Yes | Yes | No |
| VanKleef 2000^131^ | Yes | NR | NR | NR | Yes | NR | NR | NR | Yes | NR | NR |
| Terraube 2007^43^ | No | NA | NR | NR | NR | NR | NR | NR | NR | NR | NR |
| Qian 2021^44^ | Yes | Yes | NR | Yes | NR | NR | Yes | NR | Yes | NR | Yes |
| Liu 2012^140^ | NR | NR | NR | NR | NR | NR | Yes | NR | NR | NR | NR |
| Jiang 2024^81^ | NR | NR | NR | NR | NR | Yes | Yes | Yes | Yes | Yes | NR |
| Mang 2024^130^ | Yes | NR | NR | NR | NR | Yes | Yes | Yes | Yes | Yes | NR |
| South 2024^45^ | Yes | NR | NR | NR | NR | Yes | Yes | Yes | Yes | Yes | NR |
| Li 2024^82^ | Yes | Yes | NR | NR | NR | Yes | Yes | Yes | Yes | NR | NR |
| Liu 2024^113^ | Yes | NR | NR | Yes | Yes | Yes | Yes | NR | NR | NR | NR |
| Xie 2024^96^ | Yes | NR | NR | NR | Yes | Yes | Yes | NR | NR | NR | NR |
| Schellenberg 2024^166^ | Yes | Yes | NR | NR | NR | Yes | NR | NR | Yes | Yes | Yes |
| Zifkos 2024^84^ | Yes | NR | NR | NR | Yes | Yes | Yes | NR | Yes | Yes | NR |
| Jin 2024^126^ | Yes | NR | NR | NR | Yes | Yes | Yes | NR | NR | NR | NR |
| Ozawa 2024^158^ | Yes | Yes | NR | Yes | Yes | Yes | Yes | Yes | NR | NR | NR |
| Sharma 2024^114^ | NR | NR | NR | NR | NR | Yes | Yes | NR | NR | Yes | NR |
| Saeki 2023^59^ | Yes | NR | NR | Yes | Yes | NR | Yes | NR | NR | NR | Yes |
| Kim 2023^46^ | Yes | NR | NR | Yes | Yes | Yes | Yes | NR | Yes | Yes | NR |
| Oleshko 2023^93^ | NR | Yes | NR | NR | NR | NR | Yes | NR | NR | NR | NR |
| Xu 2024^83^ | Yes | Yes | NR | NR | Yes | Yes | Yes | Yes | NR | Yes | NR |
| Onodera 2023^47^ | Yes | Yes | NR | Yes | Yes | Yes | Yes | Yes | Yes | NR | NR |
| Gao 2023^48^ | Yes | NR | Yes | Yes | Yes | NR | Yes | Yes | NR | NR | Yes |
| Rossato 2023^49^ | Yes | Yes | Yes | NR | Yes | NR | Yes | Yes | NR | NR | NR |
| Ozawa 2023^60^ | Yes | Yes | Yes | Yes | Yes | Yes | Yes | Yes | Yes | Yes | Yes |
| An 2023^94^ | Yes | Yes | NR | NR | Yes | NR | Yes | Yes | NR | NR | NR |
| Lin 2022^167^ | Yes | Yes | NR | Yes | Yes | Yes | Yes | Yes | NR | Yes | Yes |
| Choi 2023^50^ | Yes | Yes | NR | Yes | Yes | Yes | Yes | Yes | NR | Yes | Yes |
| DeWilde 2023^85^ | NR | Yes | NR | NR | Yes | Yes | Yes | NR | Yes | Yes | NR |
| Ozawa 2022^141^ | Yes | Yes | Yes | Yes | Yes | NR | Yes | Yes | NR | NR | Yes |
| Fukui 2022^122^ | Yes | Yes | NR | NR | Yes | Yes | Yes | Yes | Yes | Yes | NR |
| Shi 2022^148^ | Yes | Yes | Yes | Yes | Yes | Yes | Yes | Yes | NR | NR | Yes |
| Rossato 2022^86^ | Yes | Yes | Yes | Yes | Yes | Yes | Yes | Yes | Yes | Yes | Yes |
| Courson 2022^51^ | NR | NR | Yes | Yes | Yes | NR | Yes | NR | NR | NR | Yes |
| Stivala 2022^145^ | Yes | NR | NR | Yes | Yes | Yes | Yes | NR | Yes | NR | Yes |
| Guo 2022^146^ | Yes | NR | NR | Yes | Yes | Yes | NR | NR | Yes | NR | Yes |
| Yu 2022^142^ | Yes | NR | NR | NR | Yes | Yes | Yes | NR | NR | NR | NR |
| South 2022^57^ | Yes | NR | NR | Yes | Yes | Yes | Yes | Yes | Yes | Yes | Yes |
| Zhou 2022^87^ | Yes | NR | NR | NR | Yes | Yes | Yes | Yes | NR | NR | NR |
| Meng 2021^52^ | NR | NR | NR | NR | NR | Yes | Yes | Yes | NR | NR | NR |
| Heger 2024^88^ | Yes | NR | NR | NR | Yes | Yes | NR | NR | Yes | Yes | NR |
| Xu 2023^123^ | Yes | NR | NR | NR | Yes | Yes | Yes | NR | NR | NR | NR |
| Macarthur 2024^89^ | No | NR | NR | NR | NR | Yes | NR | NR | NR | Yes | NR |
| Zelaya 2023^53^ | Yes | NR | NR | NR | NR | Yes | Yes | NR | Yes | Yes | NR |
| Li 2022^124^ | Yes | NR | NR | NR | Yes | Yes | NR | NR | Yes | NR | NR |
| Tan 2016^115^ | Yes | NR | NR | NR | Yes | NR | NR | NR | NR | NR | NR |
| Schoner 2015^149^ | Yes | NR | NR | Yes | Yes | NR | Yes | NR | NR | NR | Yes |
| Emmerechts 2012^170^ | Yes | NR | NR | NR | Yes | Yes | NR | NR | Yes | Yes | NR |
| Dmitrieva 2014^147^ | Yes | NR | Yes | Yes | Yes | NR | NR | NR | NR | NR | NR |

NR, not reported; NA, not applicable. Color is used solely for visualization and does not indicate statistical weighting or effect size.

**Supplemental material references correspond to references from manuscript, listed below:**

1. Pahwa, R., Goyal, A., & Jialal, I. (2022). Chronic Inflammation.[Updated 2022 Aug 8]. *StatPearls [Internet]. Treasure Island (FL): StatPearls Publishing*.
2. Furman D, Campisi J, Verdin E, Carrera-Bastos P, Targ S, Franceschi C, Ferrucci L, Gilroy DW, Fasano A, Miller GW, Miller AH. Chronic inflammation in the etiology of disease across the life span. Nature medicine. 2019 Dec;25(12):1822-32.
3. Peetermans M, Meyers S, Liesenborghs L, Vanhoorelbeke K, De Meyer SF, Vandenbriele C, Lox M, Hoylaerts MF, Martinod K, Jacquemin M, Vanassche T. Von Willebrand factor and ADAMTS13 impact on the outcome of Staphylococcus aureus sepsis. Journal of Thrombosis and Haemostasis. 2020 Mar 1;18(3):722-31.
4. Witsch T, Martinod K, Sorvillo N, Portier I, De Meyer SF, Wagner DD. Recombinant human ADAMTS13 treatment improves myocardial remodeling and functionality after pressure overload injury in mice. Journal of the American Heart Association. 2018 Jan 24;7(3):e007004.
5. Zhou S, Jiang S, Guo J, Xu N, Wang Q, Zhang G, Zhao L, Zhou Q, Fu X, Li L, Patzak A. ADAMTS13 protects mice against renal ischemia-reperfusion injury by reducing inflammation and improving endothelial function. American Journal of Physiology-Renal Physiology. 2019 Jan 1;316(1):F134-45.
6. Xiao J, Zhang B, Su Z, Liu Y, Shelite TR, Chang Q, Qiu Y, Bei J, Wang P, Bukreyev A, Soong L. Intracellular receptor EPAC regulates von Willebrand factor secretion from endothelial cells in a PI3K-/eNOS-dependent manner during inflammation. Journal of Biological Chemistry. 2021 Nov 1;297(5):101315.
7. Kunder CA, St John AL, Abraham SN. Mast cell modulation of the vascular and lymphatic endothelium. Blood, The Journal of the American Society of Hematology. 2011 Nov 17;118(20):5383-93.
8. Ferrero-Miliani L, Nielsen OH, Andersen PS, Girardin S. Chronic inflammation: importance of NOD2 and NALP3 in interleukin-1β generation. Clinical & Experimental Immunology. 2007 Feb;147(2):227-35.
9. Lee H, Fessler MB, Qu P, Heymann J, Kopp JB. Macrophage polarization in innate immune responses contributing to pathogenesis of chronic kidney disease. BMC nephrology. 2020 Jul 13;21(1):270.
10. Bernardo A, Ball C, Nolasco L, Moake JF, Dong JF. Effects of inflammatory cytokines on the release and cleavage of the endothelial cell-derived ultralarge von Willebrand factor multimers under flow. Blood. 2004 Jul 1;104(1):100-6. doi: 10.1182/blood-2004-01-0107. Epub 2004 Mar 16. PMID: 15026315.
11. Ono, S., Matsui, H., Noda, M. *et al.* Functional regulation of von Willebrand factor ameliorates acute ischemia-reperfusion kidney injury in mice. *Sci Rep* 9, 14453 (2019). <https://doi.org/10.1038/s41598-019-51013-2>
12. Gandhi C, Motto DG, Jensen M, Lentz SR, Chauhan AK. ADAMTS13 deficiency exacerbates VWF-dependent acute myocardial ischemia/reperfusion injury in mice. Blood. 2012 Dec 20;120(26):5224-30. doi: 10.1182/blood-2012-06-440255. Epub 2012 Sep 14. PMID: 22983446; PMCID: PMC3537314.
13. Lu, K., Liu, L., Xu, X. *et al.* ADAMTS13 ameliorates inflammatory responses in experimental autoimmune encephalomyelitis. *J Neuroinflammation* 17, 67 (2020). <https://doi.org/10.1186/s12974-020-1713-z>
14. Magallon J, Chen J, Rabbani L, Dangas G, Yang J, Bussel J, Diacovo T. Humanized mouse model of thrombosis is predictive of the clinical efficacy of antiplatelet agents. Circulation. 2011 Jan 25;123(3):319-26.
15. Mendelson AA, Lansdell C, Fox-Robichaud AE, Liaw P, Arora J, Cailhier JF, Cepinskas G, Charbonney E, Dos Santos C, Dwivedi D, Ellis CG. National Preclinical Sepsis Platform: developing a framework for accelerating innovation in Canadian sepsis research. Intensive Care Medicine Experimental. 2021 Dec;9(1):1-9.
16. Chen B, Liu H, Liu Z, Yang F. Benefits and limitations of humanized mouse models for human red blood cell-related disease research. Frontiers in Hematology. 2023 Jan 16;1:1062705.
17. Wagar LE, DiFazio RM, Davis MM. Advanced model systems and tools for basic and translational human immunology. Genome medicine. 2018 Sep 28;10(1):73.
18. Scully M, Antun A, Cataland SR, Coppo P, Dossier C, Biebuyck N, Hassenpflug WA, Kentouche K, Knöbl P, Kremer Hovinga JA, López-Fernández MF, Matsumoto M, Ortel TL, Windyga J, Bhattacharya I, Cronin M, Li H, Mellgård B, Patel M, Patwari P, Xiao S, Zhang P, Wang LT; cTTP Phase 3 Study Investigators. Recombinant ADAMTS13 in Congenital Thrombotic Thrombocytopenic Purpura. N Engl J Med. 2024 May 2;390(17):1584-1596. doi: 10.1056/NEJMoa2314793. PMID: 38692292.
19. Tricco AC, Lillie E, Zarin W, O'Brien KK, Colquhoun H, Levac D, Moher D, Peters MD, Horsley T, Weeks L, Hempel S. PRISMA extension for scoping reviews (PRISMA-ScR): checklist and explanation. Annals of internal medicine. 2018 Oct 2;169(7):467-73.
20. Covidence systematic review software, Veritas Health Innovation, Melbourne, Australia. Available at [www.covidence.org](http://www.covidence.org/).
21. Wohner N, Sebastian S, Muczynski V, Huskens D, de Laat B, de Groot PG, Lenting PJ. Osteoprotegerin modulates platelet adhesion to von Willebrand factor during release from endothelial cells. Journal of Thrombosis and Haemostasis. 2022 Mar 1;20(3):755-66.
22. Meng X, Huang W, Mo W, Shu T, Yang H, Ning H. ADAMTS-13-regulated nuclear factor E2-related factor 2 signaling inhibits ferroptosis to ameliorate cisplatin-induced acute kidney injuy: Running title: Role of ADAMTS-13 and ferroptosis in AKI. Bioengineered. 2021 Dec 20;12(2):11610-21.
23. Xiao J, Zhang B, Su Z, Liu Y, Shelite TR, Chang Q, Qiu Y, Bei J, Wang P, Bukreyev A, Soong L. Intracellular receptor EPAC regulates von Willebrand factor secretion from endothelial cells in a PI3K-/eNOS-dependent manner during inflammation. Journal of Biological Chemistry. 2021 Nov 1;297(5):101315.
24. Nguyen A, Repesse Y, Ebbo M, Allenbach Y, Benveniste O, Vallat JM, Magy L, Deshayes S, Maigne G, de Boysson H, Karnam A. IVIg increases interleukin-11 levels, which in turn contribute to increased platelets, VWF and FVIII in mice and humans. Clinical & Experimental Immunology. 2021 May;204(2):258-66.
25. Xiao J, Zhang B, Su Z, Liu Y, Shelite TR, Chang Q, Wang P, Bukreyev A, Soong L, Jin Y, Ksiazek T. EPAC regulates von Willebrand factor secretion from endothelial cells in a PI3K/eNOS-dependent manner during inflammation. bioRxiv. 2020 Sep 4:2020-09.
26. Groeneveld D, Cline-Fedewa H, Baker KS, Williams KJ, Roth RA, Mittermeier K, Lisman T, Palumbo JS, Luyendyk JP. Von Willebrand factor delays liver repair after acetaminophen-induced acute liver injury in mice. Journal of hepatology. 2020 Jan 1;72(1):146-55.
27. Chen H, Liu D, Ge L, Wang T, Ma Z, Han Y, Duan Y, Xu X, Liu W, Yuan J, Liu J. Catestatin prevents endothelial inflammation and promotes thrombus resolution in acute pulmonary embolism in mice. Bioscience reports. 2019 Nov;39(11):BSR20192236.
28. Sorvillo N, Mizurini DM, Coxon C, Martinod K, Tilvawala R, Cherpokova D, Salinger AJ, Seward RJ, Staudinger C, Weerapana E, Shapiro NI. Plasma peptidylarginine deiminase IV promotes VWF-platelet string formation and accelerates thrombosis after vessel injury. Circulation research. 2019 Aug 16;125(5):507-19.
29. Sun HJ, Chen J, Zhang H, Ni B, van Velkinburgh JC, Liu Y, Wu YZ, Yang X. Von Willebrand factor protects against acute CCl4-induced hepatotoxicity through phospho-p38 MAPK signaling pathway inhibition. Immunologic Research. 2017 Oct;65(5):1046-58.
30. Aymé G, Adam F, Legendre P, Bazaa A, Proulle V, Denis CV, Christophe OD, Lenting PJ. A novel single-domain antibody against von Willebrand factor A1 domain resolves leukocyte recruitment and vascular leakage during inflammation—brief report. Arteriosclerosis, thrombosis, and vascular biology. 2017 Sep;37(9):1736-40.
31. Zitomersky NL, Demers M, Martinod K, Gallant M, Cifuni SM, Biswas A, Snapper S, Wagner DD. ADAMTS13 deficiency worsens colitis and exogenous ADAMTS13 administration decreases colitis severity in mice. TH Open. 2017 Jun;1(01):e11-23.
32. Zhang Y, Guan L, Yu J, Zhao Z, Mao L, Li S, Zhao J. Pulmonary endothelial activation caused by extracellular histones contributes to neutrophil activation in acute respiratory distress syndrome. Respiratory research. 2016 Nov 21;17(1):155.
33. Hillgruber C, Steingräber AK, Pöppelmann B, Denis CV, Ware J, Vestweber D, Nieswandt B, Schneider SW, Goerge T. Blocking von Willebrand factor for treatment of cutaneous inflammation. Journal of Investigative Dermatology. 2014 Jan 1;134(1):77-86.
34. Amirtharaj GJ, Thangaraj KR, Kini A, Goel A, CE E, Venkatraman A, Pulimood AB, KA B, Ramachandran A. Acute liver injury induced by low dose dimethylnitrosamine alters mediators of hepatic vascular flow. Toxicology Reports. 2014 Jan 1;1:707-17.
35. Petri B, Broermann A, Li H, Khandoga AG, Zarbock A, Krombach F, Goerge T, Schneider SW, Jones C, Nieswandt B, Wild MK. von Willebrand factor promotes leukocyte extravasation. Blood, The Journal of the American Society of Hematology. 2010 Nov 25;116(22):4712-9.
36. Dieude M, Gillis MA, Theoret JF, Thorin E, Lajoie G, Levine JS, Merhi Y, Rauch J. Autoantibodies to heat shock protein 60 promote thrombus formation in a murine model of arterial thrombosis. Journal of thrombosis and haemostasis. 2009 Apr 1;7(4):710-9.
37. Noubade R, Del Rio R, McElvany B, Zachary JF, Millward JM, Wagner DD, Offner H, Blankenhorn EP, Teuscher C. von-Willebrand factor influences blood brain barrier permeability and brain inflammation in experimental allergic encephalomyelitis. The American journal of pathology. 2008 Sep 1;173(3):892-900.
38. Mimuro J, Niimura M, Kashiwakura Y, Ishiwata A, Ono T, Ohmori T, Madoiwa S, Okada K, Matsuo O, Sakata Y. Unbalanced expression of ADAMTS13 and von Willebrand factor in mouse endotoxinemia. Thrombosis research. 2008 Jan 1;122(1):91-7.
39. Rahimi S, Qian Z, Layton J, Fox-Talbot K, Baldwin III WM, Wasowska BA. Non-complement-and complement-activating antibodies synergize to cause rejection of cardiac allografts. American Journal of Transplantation. 2004 Mar 1;4(3):326-34.
40. André P, Denis CV, Ware J, Saffaripour S, Hynes RO, Ruggeri ZM, Wagner DD. Platelets adhere to and translocate on von Willebrand factor presented by endothelium in stimulated veins. Blood, The Journal of the American Society of Hematology. 2000 Nov 15;96(10):3322-8.
41. Wasowska BA, Qian Z, Cangello DL, Behrens E, Van Tran K, Layton J, Sanfilippo F, Baldwin III WM. PASSIVE TRANSFER OF ALLOANTIBODIES RESTORES ACUTE CARDIAC REJECTION IN IgKO MICE1, 2. Transplantation. 2001 Mar 27;71(6):727-36.
42. Denis CV, André P, Saffaripour S, Wagner DD. Defect in regulated secretion of P-selectin affects leukocyte recruitment in von Willebrand factor-deficient mice. Proceedings of the National Academy of Sciences. 2001 Mar 27;98(7):4072-7.
43. Terraube V, Marx I, Denis CV. Role of von Willebrand factor in tumor metastasis. Thrombosis research. 2007 Jan 1;120:S64-70.
44. Qian H, Bai Q, Yang X, Akakpo JY, Ji L, Yang L, Rülicke T, Zatloukal K, Jaeschke H, Ni HM, Ding WX. Dual roles of p62/SQSTM1 in the injury and recovery phases of acetaminophen-induced liver injury in mice. Acta Pharmaceutica Sinica B. 2021 Dec 1;11(12):3791-805.
45. South K, Roberts L, Gray A, Luka N, Strangward P, Coutts G, Smith CJ, Schiessl I, Allan SM. Inhibition of neutrophil rolling and migration by caADAMTS13 in vitro and in mouse models of thrombosis and inflammation. Biomedicine & Pharmacotherapy. 2024 Sep 1;178:117166.
46. Kim J, Qiao F, Singh AK, Won J, Singh I. Efficacies of S-nitrosoglutathione (GSNO) and GSNO reductase inhibitor in SARS-CoV-2 spike protein induced acute lung disease in mice. Frontiers in Pharmacology. 2023 Dec 8;14:1304697.
47. Onodera Y, Mitani S, Hosoda C, Takabayashi Y, Sakata A, Kawasaki R, Mori R, Ohshima C, Nishio K, Sugimoto M, Soejima K. Regulation of von Willebrand factor by ADAMTS13 ameliorates lipopolysaccharide-induced lung injury in mice. International Journal of Hematology. 2023 Dec;118(6):699-710.
48. Gao D, Zhou Z, Ma R, Wu H, Nguyen T, Liu L, Dong J. Recombinant ADAMTS-13 improves survival of mice subjected to endotoxemia. International Journal of Molecular Sciences. 2023 Jul 22;24(14):11782.
49. Rossato P, Glantschnig H, Canneva F, Schuster M, Coulibaly S, Schrenk G, Voelkel D, Dockal M, Plaimauer B, Rottensteiner H, Gritsch H. Treatment with recombinant ADAMTS13, alleviates hypoxia/reoxygenation-induced pathologies in a mouse model of human sickle cell disease. Journal of Thrombosis and Haemostasis. 2023 Feb 1;21(2):269-75.
50. Choi SJ, Dwyer CN, Rapkin L, Cormier M, Hindmarch CC, Nesbitt K, Michels A, Hopman W, Swystun LL, Lillicrap D. The mechanistic and structural role of von Willebrand factor in endotoxemia-enhanced deep vein thrombosis in mice. Journal of Thrombosis and Haemostasis. 2023 Mar 1;21(3):586-98.
51. Courson JA, Lam FW, Langlois KW, Rumbaut RE. Histone‐stimulated platelet adhesion to mouse cremaster venules in vivo is dependent on von Willebrand factor. Microcirculation. 2022 Nov;29(8):e12782.
52. Meng X, Huang W, Mo W, Shu T, Yang H, Ning H. ADAMTS-13-regulated nuclear factor E2-related factor 2 signaling inhibits ferroptosis to ameliorate cisplatin-induced acute kidney injuy: Running title: Role of ADAMTS-13 and ferroptosis in AKI. Bioengineered. 2021 Dec 20;12(2):11610-21.
53. Zelaya H, Arellano-Arriagada L, Fukuyama K, Matsumoto K, Marranzino G, Namai F, Salva S, Alvarez S, Agüero G, Kitazawa H, Villena J. Lacticaseibacillus rhamnosus CRL1505 peptidoglycan modulates the inflammation-coagulation response triggered by poly (I: C) in the respiratory tract. International Journal of Molecular Sciences. 2023 Nov 29;24(23):16907.
54. Tahir S, Wagner AH, Dietzel S, Mannell H, Pircher J, Weckbach LT, Hecker M, Pohl U. Endothelial CD40 mediates microvascular von Willebrand factor-dependent platelet adhesion inducing inflammatory venothrombosis in ADAMTS13 knockout mice. Thrombosis and Haemostasis. 2020 Mar;120(03):466-76.
55. Latifi Y, Moccetti F, Wu M, Xie A, Packwood W, Qi Y, Ozawa K, Shentu W, Brown E, Shirai T, McCarty OJ. Thrombotic microangiopathy as a cause of cardiovascular toxicity from the BCR-ABL1 tyrosine kinase inhibitor ponatinib. Blood, The Journal of the American Society of Hematology. 2019 Apr 4;133(14):1597-606.
56. Bonnefoy A, Daenens K, Feys HB, De Vos R, Vandervoort P, Vermylen J, Lawler J, Hoylaerts MF. Thrombospondin-1 controls vascular platelet recruitment and thrombus adherence in mice by protecting (sub) endothelial VWF from cleavage by ADAMTS13. Blood. 2006 Feb 1;107(3):955-64.
57. South K, Saleh O, Lemarchand E, Coutts G, Smith CJ, Schiessl I, Allan SM. Robust thrombolytic and anti-inflammatory action of a constitutively active ADAMTS13 variant in murine stroke models. Blood, The Journal of the American Society of Hematology. 2022 Mar 10;139(10):1575-87.
58. Patel KN, Soubra SH, Bellera RV, Dong JF, McMullen CA, Burns AR, Rumbaut RE. Differential role of von Willebrand factor and P-selectin on microvascular thrombosis in endotoxemia. Arteriosclerosis, thrombosis, and vascular biology. 2008 Dec 1;28(12):2225-30.
59. Saeki M, Munesue S, Higashi Y, Harashima A, Takei R, Takada S, Nakanuma S, Ohta T, Yagi S, Tajima H, Yamamoto Y. Assaying ADAMTS13 activity as a potential prognostic biomarker for sinusoidal obstruction syndrome in mice. International Journal of Molecular Sciences. 2023 Nov 15;24(22):16328.
60. Ozawa K, Packwood W, Varlamov O, Muller M, Xie A, Wu MD, Abraham-Fan RJ, López JA, Lindner JR. Elevated LDL cholesterol increases microvascular endothelial VWF and thromboinflammation after myocardial infarction. Arteriosclerosis, thrombosis, and vascular biology. 2023 Jun;43(6):1041-53.
61. Plautz WE, Haldeman SH, Dyer MR, Sperry JL, Guyette FX, Loughran PA, Alvikas J, Hassoune A, Hoteit L, Alsaadi N, Zuckerbraun BS. Reduced cleavage of von willebrand factor by ADAMTS13 is associated with microangiopathic acute kidney injury following trauma. Blood Coagulation & Fibrinolysis. 2022 Jan 1;33(1):14-24.
62. Xu L, Qiu Y, Li Y, Wei Y, Wan Y, Deng W. Tissue dynamics of von Willebrand factor characterized by a novel fluorescent protein–von Willebrand factor chimera. Journal of Thrombosis and Haemostasis. 2022 Jan 1;20(1):208-21.
63. Shentu W, Ozawa K, Nguyen TA, Wu MD, Packwood W, Xie A, Muller MA, Brown E, Hagen MW, López JA, Lindner JR. Echocardiographic molecular imaging of the effect of anticytokine therapy for atherosclerosis. Journal of the American Society of Echocardiography. 2021 Apr 1;34(4):433-42.
64. Cui W, Wu X, Feng D, Luo J, Shi Y, Guo W, Liu H, Wang Q, Wang L, Ge S, Qu Y. Acrolein induces systemic coagulopathy via autophagy-dependent secretion of von Willebrand factor in mice after traumatic brain injury. Neuroscience Bulletin. 2021 Aug;37(8):1160-75.
65. Wong SL, Goverman J, Staudinger C, Wagner DD. Recombinant human ADAMTS13 treatment and anti-NET strategies enhance skin allograft survival in mice. American Journal of Transplantation. 2020 Apr 1;20(4):1162-9.
66. Zhu JJ, Jiang ZT, Liu C, Xi YF, Wang J, Yang FF, Yao WJ, Pang W, Han LL, Zhang YH, Sun AQ. VAMP3 and SNAP23 as potential targets for preventing the disturbed flow-accelerated thrombus formation. Frontiers in Cell and Developmental Biology. 2020 Nov 5;8:576826.
67. Denorme F, Martinod K, Vandenbulcke A, Denis CV, Lenting PJ, Deckmyn H, Vanhoorelbeke K, De Meyer SF. The von Willebrand Factor A1 domain mediates thromboinflammation, aggravating ischemic stroke outcome in mice. Haematologica. 2020 Feb 27;106(3):819.
68. Ono S, Matsui H, Noda M, Kasuda S, Yada N, Yoshimoto K, Akiyama M, Miyata T, Sugimoto M, Nishio K. Functional regulation of von Willebrand factor ameliorates acute ischemia-reperfusion kidney injury in mice. Scientific Reports. 2019 Oct 8;9(1):14453.
69. Horioka K, Tanaka H, Isozaki S, Okuda K, Asari M, Shiono H, Ogawa K, Shimizu K. Hypothermia‐induced activation of the splenic platelet pool as a risk factor for thrombotic disease in a mouse model. Journal of Thrombosis and Haemostasis. 2019 Oct 1;17(10):1762-71
70. Zhou S, Jiang S, Guo J, Xu N, Wang Q, Zhang G, Zhao L, Zhou Q, Fu X, Li L, Patzak A. ADAMTS13 protects mice against renal ischemia-reperfusion injury by reducing inflammation and improving endothelial function. American Journal of Physiology-Renal Physiology. 2019 Jan 1;316(1):F134-45.
71. Wu Y, Liu W, Zhou Y, Hilton T, Zhao Z, Liu W, Wang M, Yeon J, Houck K, Thiagarajan P, Zhang F. von Willebrand factor enhances microvesicle-induced vascular leakage and coagulopathy in mice with traumatic brain injury. Blood, The Journal of the American Society of Hematology. 2018 Sep 6;132(10):1075-84.
72. Moccetti F, Brown E, Xie A, Packwood W, Qi Y, Ruggeri Z, Shentu W, Chen J, López JA, Lindner JR. Myocardial infarction produces sustained proinflammatory endothelial activation in remote arteries. Journal of the American College of Cardiology. 2018 Aug 28;72(9):1015-26.
73. Nicolay JP, Thorn V, Daniel C, Amann K, Siraskar B, Lang F, Hillgruber C, Goerge T, Hoffmann S, Gorzelanny C, Huck V. Cellular stress induces erythrocyte assembly on intravascular von Willebrand factor strings and promotes microangiopathy. Scientific reports. 2018 Jul 19;8(1):10945.
74. Urisono Y, Sakata A, Matsui H, Kasuda S, Ono S, Yoshimoto K, Nishio K, Sho M, Akiyama M, Miyata T, Okuchi K. Von willebrand factor aggravates hepatic ischemia–reperfusion injury by promoting neutrophil recruitment in mice. Thrombosis and Haemostasis. 2018 Apr;118(04):700-8.
75. Kim MG, Lim SY, Ko YS, Lee HY, Jo SK, Cho WY. ADAMTS13‐von Willebrand factor axis is involved in the pathophysiology of kidney ischaemia‐reperfusion injury. Nephrology. 2017 Nov;22(11):913-20.
76. Zhu X, Cao Y, Wei L, Cai P, Xu H, Luo H, Bai X, Lu L, Liu JR, Fan W, Zhao BQ. von Willebrand factor contributes to poor outcome in a mouse model of intracerebral haemorrhage. Scientific reports. 2016 Oct 26;6(1):35901.
77. Cai P, Luo H, Xu H, Zhu X, Xu W, Dai Y, Xiao J, Cao Y, Zhao Y, Zhao BQ, Fan W. Recombinant ADAMTS 13 attenuates brain injury after intracerebral hemorrhage. Stroke. 2015 Sep;46(9):2647-53.
78. Savchenko AS, Borissoff JI, Martinod K, De Meyer SF, Gallant M, Erpenbeck L, Brill A, Wang Y, Wagner DD. VWF-mediated leukocyte recruitment with chromatin decondensation by PAD4 increases myocardial ischemia/reperfusion injury in mice. Blood, The Journal of the American Society of Hematology. 2014 Jan 2;123(1):141-8.
79. Gandhi C, Khan MM, Lentz SR, Chauhan AK. ADAMTS13 reduces vascular inflammation and the development of early atherosclerosis in mice. Blood, The Journal of the American Society of Hematology. 2012 Mar 8;119(10):2385-91.
80. De Meyer SF, Savchenko AS, Haas MS, Schatzberg D, Carroll MC, Schiviz A, Dietrich B, Rottensteiner H, Scheiflinger F, Wagner DD. Protective anti-inflammatory effect of ADAMTS13 on myocardial ischemia/reperfusion injury in mice. Blood, The Journal of the American Society of Hematology. 2012 Dec 20;120(26):5217-23.
81. Jiang H, Hu J, He P, Wu Y, Li F, Chen Q. ADAMTS13 deficiency exacerbates neuroinflammation by targeting matrix metalloproteinase-9 in ischemic brain injury. NeuroReport. 2024 May 8;35(7):447-56.
82. Li D, Cho MS, Gonzalez‐Delgado R, Liang X, Dong JF, Cruz MA, Ma Q, Afshar‐Kharghan V. The effect of ADAMTS13 on graft‐versus‐host disease. Journal of Cellular and Molecular Medicine. 2024 Jul;28(13):e18457.
83. Xu J, He J, Zhou YL, Weng Z, Li M, Wang ZX, He Y. Von Willebrand factor promotes radiation-induced intestinal injury (RIII) development and its cleavage enzyme rhADAMTS13 protects against RIII by reducing inflammation and oxidative stress. Free Radical Biology and Medicine. 2024 Jan 1;210:1-2.
84. Zifkos K, Bochenek ML, Gogiraju R, Robert S, Pedrosa D, Kiouptsi K, Moiko K, Wagner M, Mahfoud F, Poncelet P, Münzel T. Endothelial PTP1B deletion promotes VWF exocytosis and venous thromboinflammation. Circulation Research. 2024 May 10;134(10):e93-111.
85. De Wilde M, Desender L, Tersteeg C, Vanhoorelbeke K, De Meyer SF. Spatiotemporal profile of neutrophil extracellular trap formation in a mouse model of ischemic stroke. Research and Practice in Thrombosis and Haemostasis. 2023 Jan 1;7(1):100028.
86. Rossato P, Federti E, Glantschnig H, Canneva F, Schuster M, Coulibaly S, Schrenk G, Voelkel D, Dockal M, Plaimauer B, Andolfo I. Evidence of protective effects of recombinant ADAMTS13 in a humanized model of sickle cell disease. Haematologica. 2022 Apr 21;107(11):2650.
87. Zhou S, Guo J, Liao X, Zhou Q, Qiu X, Jiang S, Xu N, Wang X, Zhao L, Hu W, Xie L. rhADAMTS13 reduces oxidative stress by cleaving VWF in ischaemia/reperfusion‐induced acute kidney injury. Acta Physiologica. 2022 Mar;234(3):e13778.
88. Heger LA, Schommer N, Van Bruggen S, Sheehy CE, Chan W, Wagner DD. Neutrophil NLRP3 promotes cardiac injury following acute myocardial infarction through IL-1β production, VWF release and NET deposition in the myocardium. Scientific Reports. 2024 Jun 24;14(1):14524.
89. MacArthur TA, Goswami J, Navarro SM, Spears GM, Bailey KR, Thompson R, Dong JF, Kozar RA, Auton MT, Knight J, Park MS. A murine multiple-injury model for the study of thromboinflammation. Journal of Trauma and Acute Care Surgery. 2024 Feb 1;96(2):203-8.
90. Fujioka M, Nakano T, Hayakawa K, Irie K, Akitake Y, Sakamoto Y, Mishima K, Muroi C, Yonekawa Y, Banno F, Kokame K. ADAMTS13 gene deletion enhances plasma high-mobility group box1 elevation and neuroinflammation in brain ischemia–reperfusion injury. Neurological sciences. 2012 Oct;33(5):1107-15.
91. Fujioka M, Hayakawa K, Mishima K, Kunizawa A, Irie K, Higuchi S, Nakano T, Muroi C, Fukushima H, Sugimoto M, Banno F. ADAMTS13 gene deletion aggravates ischemic brain damage: a possible neuroprotective role of ADAMTS13 by ameliorating postischemic hypoperfusion. Blood, The Journal of the American Society of Hematology. 2010 Feb 25;115(8):1650-3.
92. Bayat B, Tjahjono Y, Berghöfer H, Werth S, Deckmyn H, De Meyer SF, Sachs UJ, Santoso S. Choline transporter-like protein-2: new von Willebrand factor–binding partner involved in antibody-mediated neutrophil activation and transfusion-related acute lung injury. Arteriosclerosis, Thrombosis, and Vascular Biology. 2015 Jul;35(7):1616-22.
93. Oleshko O, Vollack-Hesse N, Tiede A, Hegermann J, Curth U, Werwitzke S. von Willebrand factor modulates immune complexes and the recall response against factor VIII in a murine hemophilia A model. Blood Advances. 2023 Nov 14;7(21):6771-81.
94. An R, Man Y, Cheng K, Zhang T, Chen C, Wang F, Abdulla F, Kucukal E, Wulftange WJ, Goreke U, Bode A. Sickle red blood cell‐derived extracellular vesicles activate endothelial cells and enhance sickle red cell adhesion mediated by von Willebrand factor. British journal of haematology. 2023 May;201(3):552-63.
95. Chauhan AK, Kisucka J, Brill A, Walsh MT, Scheiflinger F, Wagner DD. ADAMTS13: a new link between thrombosis and inflammation. The Journal of experimental medicine. 2008 Sep 1;205(9):2065-74.
96. Xie Y, Lv H, Chen D, Huang P, Wu S, Shi H, Zhao Q, Wang R. Recombinant human thrombopoietin in alleviating endothelial cell injury in sepsis. Journal of Intensive Medicine. 2024 Jul 25;4(03):384-92.
97. Na M, Hu Z, Mohammad M, Stroparo MD, Ali A, Fei Y, Jarneborn A, Verhamme P, Schneewind O, Missiakas D, Jin T. The expression of von Willebrand factor-binding protein determines joint-invading capacity of Staphylococcus aureus, a Core mechanism of septic arthritis. MBio. 2020 Dec 22;11(6):10-128.
98. Peetermans M, Meyers S, Liesenborghs L, Vanhoorelbeke K, De Meyer SF, Vandenbriele C, Lox M, Hoylaerts MF, Martinod K, Jacquemin M, Vanassche T. Von Willebrand factor and ADAMTS13 impact on the outcome of Staphylococcus aureus sepsis. Journal of Thrombosis and Haemostasis. 2020 Mar 1;18(3):722-31.
99. Liesenborghs L, Peetermans M, Claes J, Veloso TR, Vandenbriele C, Criel M, Lox M, Peetermans WE, Heilbronner S, de Groot PG, Vanassche T. Shear-resistant binding to von Willebrand factor allows Staphylococcus lugdunensis to adhere to the cardiac valves and initiate endocarditis. The Journal of infectious diseases. 2016 Apr 1;213(7):1148-56.
100. Claes J, Vanassche T, Peetermans M, Liesenborghs L, Vandenbriele C, Vanhoorelbeke K, Missiakas D, Schneewind O, Hoylaerts MF, Heying R, Verhamme P. Adhesion of Staphylococcus aureus to the vessel wall under flow is mediated by von Willebrand factor–binding protein. Blood, The Journal of the American Society of Hematology. 2014 Sep 4;124(10):1669-76.
101. Pappelbaum KI, Gorzelanny C, Grässle S, Suckau J, Laschke MW, Bischoff M, Bauer C, Schorpp-Kistner M, Weidenmaier C, Schneppenheim R, Obser T. Ultralarge von Willebrand factor fibers mediate luminal Staphylococcus aureus adhesion to an intact endothelial cell layer under shear stress. Circulation. 2013 Jul 2;128(1):50-9.
102. Huang J, Motto DG, Bundle DR, Sadler JE. Shiga toxin B subunits induce VWF secretion by human endothelial cells and thrombotic microangiopathy in ADAMTS13-deficient mice. Blood, The Journal of the American Society of Hematology. 2010 Nov 4;116(18):3653-9.
103. Liesenborghs L, Meyers S, Lox M, Criel M, Claes J, Peetermans M, Trenson S, Vande Velde G, Vanden Berghe P, Baatsen P, Missiakas D. Staphylococcus aureus endocarditis: distinct mechanisms of bacterial adhesion to damaged and inflamed heart valves. European heart journal. 2019 Oct 14;40(39):3248-59.
104. Claes J, Liesenborghs L, Peetermans M, Veloso TR, Missiakas D, Schneewind O, Mancini S, Entenza JM, Hoylaerts MF, Heying R, Verhamme P. Clumping factor A, von Willebrand factor‐binding protein and von Willebrand factor anchor Staphylococcus aureus to the vessel wall. Journal of Thrombosis and Haemostasis. 2017 May 1;15(5):1009-19.
105. Chung MC, Popova TG, Jorgensen SC, Dong L, Chandhoke V, Bailey CL, Popov SG. Degradation of circulating von Willebrand factor and its regulator ADAMTS13 implicates secreted Bacillus anthracis metalloproteases in anthrax consumptive coagulopathy. Journal of Biological Chemistry. 2008 Apr 11;283(15):9531-42.
106. Chauhan AK, Walsh MT, Zhu G, Ginsburg D, Wagner DD, Motto DG. The combined roles of ADAMTS13 and VWF in murine models of TTP, endotoxemia, and thrombosis. Blood, The Journal of the American Society of Hematology. 2008 Apr 1;111(7):3452-7.
107. O’regan N, Gegenbauer K, O’Sullivan JM, Maleki S, Brophy TM, Dalton N, Chion A, Fallon PG, Grau GE, Budde U, Smith OP. A novel role for von Willebrand factor in the pathogenesis of experimental cerebral malaria. Blood, The Journal of the American Society of Hematology. 2016 Mar 3;127(9):1192-201.
108. Kraisin S, Verhenne S, Pham TT, Martinod K, Tersteeg C, Vandeputte N, Deckmyn H, Vanhoorelbeke K, Van den Steen PE, De Meyer SF. von Willebrand factor in experimental malaria‐associated acute respiratory distress syndrome. Journal of Thrombosis and Haemostasis. 2019 Aug 1;17(8):1372-83.
109. Kraisin S, Martinod K, Desender L, Pareyn I, Verhenne S, Deckmyn H, Vanhoorelbeke K, Van den Steen PE, De Meyer SF. von Willebrand factor increases in experimental cerebral malaria but is not essential for late‐stage pathogenesis in mice. Journal of Thrombosis and Haemostasis. 2020 Sep 1;18(9):2377-90.
110. Alflen A, Prüfer S, Ebner K, Reuter S, Aranda Lopez P, Scharrer I, Banno F, Stassen M, Schild H, Jurk K, Bosmann M. ADAMTS-13 regulates neutrophil recruitment in a mouse model of invasive pulmonary aspergillosis. Scientific reports. 2017 Aug 3;7(1):7184.
111. Patel KN, Soubra SH, Lam FW, Rodriguez MA, Rumbaut RE. Polymicrobial sepsis and endotoxemia promote microvascular thrombosis via distinct mechanisms. Journal of Thrombosis and Haemostasis. 2010 Jun 1;8(6):1403-9.
112. Lerolle N, Lardé CD, Badirou I, Motto DG, Hill G, Bruneval P, Diehl JL, Denis CV, Baruch D. von Willebrand factor is a major determinant of ADAMTS-13 decrease during mouse sepsis induced by cecum ligation and puncture. Journal of Thrombosis and Haemostasis. 2009 May 1;7(5):843-50.
113. Liu YS, Chen WL, Zeng YW, Li ZH, Zheng HL, Pan N, Zhao LY, Wang S, Chen SH, Jiang MH, Jin CC. Isaridin E Protects against Sepsis by Inhibiting Von Willebrand Factor-Induced Endothelial Hyperpermeability and Platelet–Endothelium Interaction. Marine Drugs. 2024 Jun 16;22(6):283.
114. Sharma N, Chen A, Heinen L, Liu R, Dwivedi DJ, Zhou J, Lalu MM, Mendelson AA, McDonald B, Kretz CA, Fox-Robichaud AE. Impact of age on the host response to sepsis in a murine model of fecal-induced peritonitis. Intensive Care Medicine Experimental. 2024 Mar 8;12(1):28.
115. Tan L, Huang Y, Pan X, Quan S, Xu S, Li D, Song L, Zhang X, Chen W, Pan J. Administration of bone marrow stromal cells in sepsis attenuates sepsis-related coagulopathy. Annals of Medicine. 2016 May 18;48(4):235-45.
116. Jin X, Jin C, Nakamura K, Jin T, Xin M, Wan Y, Yue X, Jin S, Wang H, Inoue A, Nan Y. Increased dipeptidyl peptidase-4 accelerates chronic stress-related thrombosis in a mouse carotid artery model. Journal of Hypertension. 2020 Aug 1;38(8):1504-13.
117. Lu K, Liu L, Xu X, Zhao F, Deng J, Tang X, Wang X, Zhao BQ, Zhang X, Zhao Y. ADAMTS13 ameliorates inflammatory responses in experimental autoimmune encephalomyelitis. Journal of Neuroinflammation. 2020 Feb 19;17(1):67.
118. Suraj J, Kurpińska A, Zakrzewska A, Sternak M, Stojak M, Jasztal A, Walczak M, Chlopicki S. Early and late endothelial response in breast cancer metastasis in mice: simultaneous quantification of endothelial biomarkers using a mass spectrometry-based method. Disease models & mechanisms. 2019 Mar 1;12(3):dmm036269.
119. Dhanesha N, Doddapattar P, Chorawala MR, Nayak MK, Kokame K, Staber JM, Lentz SR, Chauhan AK. ADAMTS13 retards progression of diabetic nephropathy by inhibiting intrarenal thrombosis in mice. Arteriosclerosis, thrombosis, and vascular biology. 2017 Jul;37(7):1332-8.
120. Adam F, Casari C, Prévost N, Kauskot A, Loubière C, Legendre P, Repérant C, Baruch D, Rosa JP, Bryckaert M, de Groot PG. A genetically-engineered von Willebrand disease type 2B mouse model displays defects in hemostasis and inflammation. Scientific reports. 2016 May 23;6(1):26306.
121. Denis CV, André P, Saffaripour S, Wagner DD. Defect in regulated secretion of P-selectin affects leukocyte recruitment in von Willebrand factor-deficient mice. Proceedings of the National Academy of Sciences. 2001 Mar 27;98(7):4072-7.
122. Fukui S, Gutch S, Fukui S, Chu L, Wagner DD. Anti‐inflammatory protective effect of ADAMTS‐13 in murine arthritis models. Journal of Thrombosis and Haemostasis. 2022 Oct 1;20(10):2386-93.
123. Xu S, Piao L, Wan Y, Huang Z, Meng X, Inoue A, Wang H, Yue X, Jin X, Shi GP, Kuzuya M. CTSS modulates stress-related carotid artery thrombosis in a mouse FeCl3 model. Arteriosclerosis, Thrombosis, and Vascular Biology. 2023 Jul;43(7):e238-53.
124. Li K, Wang Q, Lv Q, Guo K, Han L, Duan P, Deng Y, Bian H. Wenyang Huazhuo Tongluo formula alleviates pulmonary vascular injury and downregulates HIF-1α in bleomycin-induced systemic sclerosis mouse model. BMC Complementary Medicine and Therapies. 2022 Jun 22;22(1):167.
125. Xiang Y, Cheng J, Wang D, Hu X, Xie Y, Stitham J, Atteya G, Du J, Tang WH, Lee SH, Leslie K. Hyperglycemia repression of miR-24 coordinately upregulates endothelial cell expression and secretion of von Willebrand factor. Blood, The Journal of the American Society of Hematology. 2015 May 28;125(22):3377-87.
126. Jin X, Yue X, Huang Z, Meng X, Xu S, Wu Y, Wan Y, Inoue A, Narisawa M, Hu L, Shi GP. Cathepsin K deficiency prevented stress-related thrombosis in a mouse FeCl3 model. Cellular and Molecular Life Sciences. 2024 Dec;81(1):205.
127. Zhou S, Guo J, Zhao L, Liao Y, Zhou Q, Cui Y, Hu W, Chen J, Ren X, Wei Q, Jiang S. ADAMTS13 inhibits oxidative stress and ameliorates progressive chronic kidney disease following ischaemia/reperfusion injury. Acta Physiologica. 2021 Mar;231(3):e13586.
128. Witsch T, Martinod K, Sorvillo N, Portier I, De Meyer SF, Wagner DD. Recombinant human ADAMTS13 treatment improves myocardial remodeling and functionality after pressure overload injury in mice. Journal of the American Heart Association. 2018 Jan 24;7(3):e007004.
129. Qin F, Impeduglia T, Schaffer P, Dardik H. Overexpression of von Willebrand factor is an independent risk factor for pathogenesis of intimal hyperplasia: preliminary studies. Journal of vascular surgery. 2003 Feb 1;37(2):433-9.
130. Mang G, Chen J, Sun P, Ma R, Du J, Wang X, Cui J, Yang M, Tong Z, Yan X, Wang D. Von Willebrand factor exacerbates heart failure through formation of neutrophil extracellular traps. European Heart Journal. 2024 Oct 1;45(37):3853-67
131. van Kleef E, Verheij M, Poele HT, Oussoren Y, Dewit L, Stewart F. In vitro and in vivo expression of endothelial von Willebrand factor and leukocyte accumulation after fractionated irradiation. Radiation research. 2000 Oct 1;154(4):375-81.
132. Ozawa K, Muller MA, Varlamov O, Tavori H, Packwood W, Mueller PA, Xie A, Ruggeri Z, Chung D, López JA, Lindner JR. Proteolysis of von Willebrand factor influences inflammatory endothelial activation and vascular compliance in atherosclerosis. Basic to Translational Science. 2020 Oct 1;5(10):1017-28.
133. Michels A, Dwyer CN, Mewburn J, Nesbitt K, Kawecki C, Lenting P, Swystun LL, Lillicrap D. von Willebrand factor is a critical mediator of deep vein thrombosis in a mouse model of diet-induced obesity. Arteriosclerosis, Thrombosis, and Vascular Biology. 2020 Dec;40(12):2860-74.
134. Yang J, Lu Y, Lou X, Wang J, Yu H, Bao Z, Wang H. Von Willebrand factor deficiency improves hepatic steatosis, insulin resistance, and inflammation in mice fed high‐fat diet. Obesity. 2020 Apr;28(4):756-64.
135. Doddapattar P, Dhanesha N, Chorawala MR, Tinsman C, Jain M, Nayak MK, Staber JM, Chauhan AK. Endothelial cell–derived von Willebrand factor, but not platelet-derived, promotes atherosclerosis in apolipoprotein E–deficient mice. Arteriosclerosis, thrombosis, and vascular biology. 2018 Mar;38(3):520-8.
136. Geys L, Bauters D, Roose E, Tersteeg C, Vanhoorelbeke K, Hoylaerts MF, Lijnen RH, Scroyen I. ADAMTS13 deficiency promotes microthrombosis in a murine model of diet-induced liver steatosis. Thrombosis and Haemostasis. 2017 Aug;26(01):19-26.
137. Hung YC, Yang HT, Yin MC. Asiatic acid and maslinic acid protected heart via anti-glycative and anti-coagulatory activities in diabetic mice. Food & function. 2015;6(9):2967-74.
138. Iwaki T, Sandoval-Cooper MJ, Brechmann M, Ploplis VA, Castellino FJ. A fibrinogen deficiency accelerates the initiation of LDL cholesterol–driven atherosclerosis via thrombin generation and platelet activation in genetically predisposed mice. Blood. 2006 May 15;107(10):3883-91.
139. Methia N, André P, Denis CV, Economopoulos M, Wagner DD. Localized reduction of atherosclerosis in von Willebrand factor–deficient mice. Blood, The Journal of the American Society of Hematology. 2001 Sep 1;98(5):1424-8.
140. Liu MY, Zhou Z, Ma R, Tao Z, Choi H, Bergeron AL, Wu H, Dong JF. Gender-dependent up-regulation of ADAMTS-13 in mice with obesity and hypercholesterolemia. Thrombosis research. 2012 Apr 1;129(4):536-9.
141. Ozawa K, Muller MA, Varlamov O, Hagen MW, Packwood W, Morgan TK, Xie A, López CS, Chung D, Chen J, López JA. Reduced proteolytic cleavage of von Willebrand factor leads to aortic valve stenosis and load-dependent ventricular remodeling. Basic to Translational Science. 2022 Jul 1;7(7):642-55.
142. Yu GH, Fang Y. Resveratrol attenuates atherosclerotic endothelial injury through the Pin1/Notch1 pathway. Toxicology and Applied Pharmacology. 2022 Jul 1;446:116047.
143. Jin SY, Tohyama J, Bauer RC, Cao NN, Rader DJ, Zheng XL. Genetic ablation of Adamts13 gene dramatically accelerates the formation of early atherosclerosis in a murine model. Arteriosclerosis, thrombosis, and vascular biology. 2012 Aug;32(8):1817-23.
144. Gandhi C, Khan MM, Lentz SR, Chauhan AK. ADAMTS13 reduces vascular inflammation and the development of early atherosclerosis in mice. Blood, The Journal of the American Society of Hematology. 2012 Mar 8;119(10):2385-91.
145. Stivala S, Gobbato S, Bonetti N, Camici GG, Lüscher TF, Beer JH. Dietary alpha‐linolenic acid reduces platelet activation and collagen‐mediated cell adhesion in sickle cell disease mice. Journal Of Thrombosis And Haemostasis. 2022 Feb 1;20(2):375-86.
146. Guo S, Zhang S, Chen K, Chen X, Hu F. Effects of diagnostic ultrasound with cRGD-microbubbles on simultaneous detection and treatment of atherosclerotic plaque in ApoE−/− mice. Frontiers in Cardiovascular Medicine. 2022 Jul 22;9:946557
147. Dmitrieva NI, Burg MB. Secretion of von Willebrand factor by endothelial cells links sodium to hypercoagulability and thrombosis. Proceedings of the National Academy of Sciences. 2014 Apr 29;111(17):6485-90.
148. Shi Q, Fahs SA, Mattson JG, Yu H, Perry CL, Morateck PA, Schroeder JA, Rapten J, Weiler H, Montgomery RR. A novel mouse model of type 2N VWD was developed by CRISPR/Cas9 gene editing and recapitulates human type 2N VWD. Blood Advances. 2022 May 10;6(9):2778-90.
149. Schoner A, Tyrrell C, Wu M, Gelow JM, Hayes AA, Lindner JR, Thornburg KL, Hasan W. Endocardial endothelial dysfunction progressively disrupts initially anti then pro-thrombotic pathways in heart failure mice. PloS one. 2015 Nov 13;10(11):e0142940.
150. Kiouptsi K, Grill A, Mann A, Döhrmann M, Lillich M, Jäckel S, Malinarich F, Formes H, Manukyan D, Subramaniam S, Khandagale A. Mice deficient in the anti-haemophilic coagulation factor VIII show increased von Willebrand factor plasma levels. PLoS One. 2017 Aug 24;12(8):e0183590.
151. Chen J, Schroeder JA, Luo X, Shi Q. The impact of von Willebrand factor on factor VIII memory immune responses. Blood advances. 2017 Aug 22;1(19):1565-74.
152. Shim CY, Liu YN, Atkinson T, Xie A, Foster T, Davidson BP, Treible M, Qi Y, López JA, Munday A, Ruggeri Z. Molecular imaging of platelet–endothelial interactions and endothelial von Willebrand factor in early and mid-stage atherosclerosis. Circulation: Cardiovascular Imaging. 2015 Jul;8(7):e002765.
153. Rhieu BH, Epperly MW, Cao S, Franicola D, Shields D, Goff J, Wang H, Greenberger JS. Increased hematopoiesis in long-term bone marrow cultures and reduced irradiation-induced pulmonary fibrosis in Von Willebrand factor homologous deletion recombinant mice. in vivo. 2014 Jul 1;28(4):449-56.
154. Delignat S, Repessé Y, Navarrete AM, Meslier Y, Gupta N, Christophe OD, Kaveri SV, Lacroix‐Desmazes S. Immunoprotective effect of von Willebrand factor towards therapeutic factor VIII in experimental haemophilia A. Haemophilia. 2012 Mar;18(2):248-54.
155. McCarty OJ, Conley RB, Shentu W, Tormoen GW, Zha D, Xie A, Qi Y, Zhao Y, Carr C, Belcik T, Keene DR. Molecular imaging of activated von Willebrand factor to detect high-risk atherosclerotic phenotype. JACC: Cardiovascular Imaging. 2010 Sep;3(9):947-55.
156. Delignat S, Dasgupta S, André S, Navarrete AM, Kaveri SV, Bayry J, André MH, Chtourou S, Tellier Z, Lacroix-Desmazes S. Comparison of the immunogenicity of different therapeutic preparations of human factor VIII in the murine model of hemophilia A. haematologica. 2007 Oct 1;92(10):1423-6.
157. Kallas A, Kuuse S, Maimets T, Pooga M. von Willebrand factor and transforming growth factor-beta modulate immune response against coagulation factor VIII in FVIII-deficient mice. Thrombosis research. 2007 Jan 1;120(6):911-9.
158. Ozawa K, Packwood W, Muller MA, Qi Y, Xie A, Varlamov O, McCarty OJ, Chung D, López JA, Lindner JR. Removal of endothelial surface-associated von villebrand factor suppresses accelerate datherosclerosis after myocardial infarction. Journal of translational medicine. 2024 May 1;22(1):412.
159. Bauer AT, Suckau J, Frank K, Desch A, Goertz L, Wagner AH, Hecker M, Goerge T, Umansky L, Beckhove P, Utikal J. von Willebrand factor fibers promote cancer-associated platelet aggregation in malignant melanoma of mice and humans. Blood, The Journal of the American Society of Hematology. 2015 May 14;125(20):3153-63.
160. Espirito Santo SM, Pires NM, Boesten LS, Gerritsen G, Bovenschen N, Van Dijk KW, Jukema JW, Princen HM, Bensadoun A, Li WP, Herz J. Hepatic low-density lipoprotein receptor–related protein deficiency in mice increases atherosclerosis independent of plasma cholesterol. Blood. 2004 May 15;103(10):3777-82.
161. Kang JJ, Kaissarian NM, Desch KC, Kelly RJ, Shu L, Bodary PF, Shayman JA. α-galactosidase A deficiency promotes von Willebrand factor secretion in models of Fabry disease. Kidney international. 2019 Jan 1;95(1):149-59.
162. Mojiri A, Nakhaii-Nejad M, Phan WL, Kulak S, Radziwon-Balicka A, Jurasz P, Michelakis E, Jahroudi N. Hypoxia results in upregulation and de novo activation of von Willebrand factor expression in lung endothelial cells. Arteriosclerosis, thrombosis, and vascular biology. 2013 Jun;33(6):1329-38.
163. Dincel GC, Atmaca HT. Increased expressions of ADAMTS‐13 and apoptosis contribute to neuropathology during Toxoplasma gondii encephalitis in mice. Neuropathology. 2016 Jun;36(3):211-26.
164. Poole LG, Fournier AK, Cline-Fedewa HM, Kopec AK, Luyendyk JP, Groeneveld DJ. Von Willebrand factor exerts hepatoprotective effects in acute but not chronic cholestatic liver injury in mice. Toxicology. 2021 Nov 1;463:152968.
165. Joshi N, Kopec AK, Ray JL, Cline-Fedewa H, Groeneveld DJ, Lisman T, Luyendyk JP. Von Willebrand factor deficiency reduces liver fibrosis in mice. Toxicology and applied pharmacology. 2017 Aug 1;328:54-9.
166. Schellenberg C, Lagrange J, Ahmed MU, Arnone D, Campoli P, Louis H, Touly N, Caron B, Plénat F, Perrin J, Lenting PJ. The role of platelets and von Willebrand factor in the procoagulant phenotype of inflammatory bowel disease. Journal of Crohn's and Colitis. 2024 May 1;18(5):751-61.
167. Li K, Wang Q, Lv Q, Guo K, Han L, Duan P, Deng Y, Bian H. Wenyang Huazhuo Tongluo formula alleviates pulmonary vascular injury and downregulates HIF-1α in bleomycin-induced systemic sclerosis mouse model. BMC Complementary Medicine and Therapies. 2022 Jun 22;22(1):167.
168. Michels A, Albánez S, Mewburn J, Nesbitt K, Gould TJ, Liaw PC, James PD, Swystun LL, Lillicrap D. Histones link inflammation and thrombosis through the induction of Weibel–Palade body exocytosis. Journal of Thrombosis and Haemostasis. 2016 Nov 1;14(11):2274-86.
169. Ostertag EM, Bdeir K, Kacir S, Thiboutot M, Gulendran G, Yunk L, Hayes VM, Motto DG, Poncz M, Zheng XL, Cines DB. ADAMTS13 autoantibodies cloned from patients with acquired thrombotic thrombocytopenic purpura: 2. Pathogenicity in an animal model. Transfusion. 2016 Jul;56(7):1775-85.
170. Emmerechts J, De Vooght V, Haenen S, Loyen S, Hemmeryckx B, Vanoirbeek JA, Hoet PH, Nemery B, Hoylaerts MF. Thrombogenic changes in young and old mice upon subchronic exposure to air pollution in an urban roadside tunnel. Thrombosis and haemostasis. 2012;108(10):756-68.
171. Gragnano F, Sperlongano S, Golia E, Natale F, Bianchi R, Crisci M, Fimiani F, Pariggiano I, Diana V, Carbone A, Cesaro A. The role of von Willebrand factor in vascular inflammation: from pathogenesis to targeted therapy. Mediators of inflammation. 2017;2017(1):5620314.
172. Diaz JA, Obi AT, Myers Jr DD, Wrobleski SK, Henke PK, Mackman N, Wakefield TW. Critical review of mouse models of venous thrombosis. Arteriosclerosis, thrombosis, and vascular biology. 2012 Mar;32(3):556-62.
173. Sharma, R., Kapila, R., Ul Haq, M. R., Salingati, V., Kapasiya, M., & Kapila, S. (2014). Age-associated aberrations in mouse cellular and humoral immune responses. Aging Clinical and Experimental Research.
174. Ameho, C. K., Adjei, A. A., Yamauchi, K., Harrison, E. K., Kulkarni, A. D., Sato, S., & Yamamoto, S. (1997). Modulation of age-related changes in immune functions of protein-deficient senescence-accelerated mice by dietary nucleoside-nucleotide mixture supplementation. British Journal of Nutrition.
175. Biguzzi E, Castelli F, Lijfering WM, Cannegieter SC, Eikenboom J, Rosendaal FR, van Hylckama Vlieg A. Rise of levels of von Willebrand factor and factor VIII with age: role of genetic and acquired risk factors. Thrombosis Research. 2021 Jan 1;197:172-8.
176. Poston JN, Kruse-Jarres R. How I treat von Willebrand disorders in older adults. Blood. 2024 Jan 18;143(3):197-204.
177. Goudemand J, Susen S. von Willebrand factor: aging is better?. Journal of Thrombosis and Haemostasis. 2023 Dec 1;21(12):3357-9.
178. Klein, S. L., & Flanagan, K. L. (2016). Sex differences in immune responses. Nature Reviews Immunology.
179. Rossetti, A. C., Paladini, M. S., Trepci, A., Mallien, A., Riva, M. A., Gass, P., & Molteni, R. (2019). Differential neuroinflammatory response in male and female mice: A role for BDNF. Frontiers in Molecular Neuroscience.
180. Harrison RL, McKee PA. Estrogen stimulates von Willebrand factor production by cultured endothelial cells.
181. Salem ML. Estrogen, a double-edged sword: modulation of TH1-and TH2-mediated inflammations by differential regulation of TH1/TH2 cytokine production. Current Drug Targets-Inflammation & Allergy. 2004 Mar 1;3(1):97-104.
182. Kay E, Gomez-Garcia L, Woodfin A, Scotland RS, Whiteford JR. Sexual dimorphisms in leukocyte trafficking in a mouse peritonitis model. Journal of Leucocyte Biology. 2015 Nov;98(5):805-17.
183. Seemann, S., Zohles, F., & Lupp, A. (2017). Comprehensive comparison of three different animal models for systemic inflammation. Journal of Biomedical Science.
184. Kim, A., Fung, E., Parikh, S. G., Valore, E. V., Gabayan, V., Nemeth, E., & Ganz, T. (2014). A mouse model of anemia of inflammation: complex pathogenesis with partial dependence on hepcidin. Blood.
185. Libby, P., & Hansson, G. K. (2015). Inflammation and atherosclerosis: from pathophysiology to practice. Journal of the American College of Cardiology.
186. Wen, C., Chen, D., Zhong, R., & Peng, X. (2024). Animal models of inflammatory bowel disease: category and evaluation indexes. Gastroenterology Report.
187. Chen J, Chung DW. Inflammation, von Willebrand factor, and ADAMTS13. Blood, The Journal of the American Society of Hematology. 2018 Jul 12;132(2):141-7.
188. Michels, A., Dwyer, C. N., Mewburn, J., Nesbitt, K., Kawecki, C., Lenting, P. J., Swystun, L. L., & Lillicrap, D. (2020). von Willebrand Factor Is a Critical Mediator of Deep Vein Thrombosis in a Mouse Model of Diet-Induced Obesity. Arteriosclerosis, Thrombosis, and Vascular Biology.
189. Kopec, A. K., Abrahams, S. R., Thornton, S., Palumbo, J. S., Mullins, E. S., Divanovic, S., Weiler, H., Owens, A. P. III, Mackman, N., Goss, A., van Ryn, J., Luyendyk, J. P., & Flick, M. J. (2017). Thrombin promotes diet-induced obesity through fibrin-driven inflammation. Journal of Clinical Investigation.
190. Somann JP, Wasilczuk KM, Neihouser KV, Sturgis J, Albors GO, Robinson JP, Powley TL, Irazoqui PP. Characterization of plasma cytokine response to intraperitoneally administered LPS & subdiaphragmatic branch vagus nerve stimulation in rat model. PLoS One. 2019 Mar 28;14(3):e0214317.
191. Kosyreva AM, Makarova OV, Kakturskiy LV, Mikhailova LP, Boltovskaya MN, Rogov KA. Sex differences of inflammation in target organs, induced by intraperitoneal injection of lipopolysaccharide, depend on its dose. Journal of Inflammation Research. 2018 Nov 8:431-45.
192. Brooks D, Barr LC, Wiscombe S, McAuley DF, Simpson AJ, Rostron AJ. Human lipopolysaccharide models provide mechanistic and therapeutic insights into systemic and pulmonary inflammation. European Respiratory Journal. 2020 Jul 30;56(1).
193. Fullerton JN, Segre E, De Maeyer RP, Maini AA, Gilroy DW. Intravenous endotoxin challenge in healthy humans: an experimental platform to investigate and modulate systemic inflammation. Journal of visualized experiments: JoVE. 2016 May 16(111):53913.
194. Olfert, E. D., & Godson, D. L. (2000). Humane Endpoints for Infectious Disease Animal Models. ILAR Journal
195. Du Sert NP, Ahluwalia A, Alam S, Avey MT, Baker M, Browne WJ, Clark A, Cuthill IC, Dirnagl U, Emerson M, Garner P. Reporting animal research: Explanation and elaboration for the ARRIVE guidelines 2.0. PLoS biology. 2020 Jul 14;18(7):e3000411.
196. Ahmadi-Noorbakhsh S, Farajli Abbasi M, Ghasemi M, Bayat G, Davoodian N, Sharif-Paghaleh E, Poormoosavi SM, Rafizadeh M, Maleki M, Shirzad-Aski H, Kargar Jahromi H. Anesthesia and analgesia for common research models of adult mice. Laboratory animal research. 2022 Dec 13;38(1):40.
197. Han, Y., Itenberg, S. A., Wu, X., & Xiao, H. (2022). Guidelines for inflammation models in mice for food components. Experimental and Molecular Medicine.
198. Knuth MM, Campos CV, Smith K, Hutchins EK, Lewis S, York M, Coghill LM, Franklin C, MacFarlane AJ, Ericsson AC, Magnuson T. Timing of standard chow exposure determines the variability of mouse phenotypic outcomes and gut microbiota profile. Lab animal. 2025 Jan;54(1):24-36.
199. Morrison KE, Jašarević E, Howard CD, Bale TL. It's the fiber, not the fat: significant effects of dietary challenge on the gut microbiome. Microbiome. 2020 Feb 11;8(1):15.
200. Zhao L, Wei J, Yue B. Brief introduction in phenotypic and genetic differences of C57BL/6 and BALB/c mice substrains. Animal Models and Experimental Medicine. 2025 Sep;8(9):1628-34.
201. Tillgren, S. M., Nieto-Fontarigo, J. J., Cerps, S., Ramu, S., Menzel, M., Mahmutovic Persson, I., Meissner, A., Akbarshahi, H., & Uller, L. (2023). C57Bl/6N mice have an attenuated lung inflammatory response to dsRNA compared to C57Bl/6J and BALB/c mice. Journal of Inflammation, 20, Article number: 6.
